# Functional connectome signature of general psychopathology in middle-aged and older adults: Evidence from multi-cohort, multi-ethnic analyses

**DOI:** 10.1162/IMAG.a.1308

**Published:** 2026-07-20

**Authors:** Thuan Tinh Nguyen, Kwun Kei Ng, Voon Hao Lew, Janice Jue Xin Koi, Wen Liang Loh, Woon-Puay Koh, Juan Helen Zhou

**Affiliations:** Centre for Sleep and Cognition & Centre for Translational Magnetic Resonance Research, Yong Loo Lin School of Medicine, National University of Singapore, Singapore, Singapore; Healthy Longevity & Human Potential Translational Research Program and Department of Medicine, Yong Loo Lin School of Medicine, National University of Singapore, Singapore; Institute for Human Development and Potential (IHDP), Agency for Science, Technology and Research (A*STAR), Singapore, Singapore; Department of Electrical and Computer Engineering & Integrative Sciences and Engineering Programme (ISEP), NUS Graduate School, National University of Singapore, Singapore, Singapore

**Keywords:** functional connectivity, psychopathology, transdiagnostic, middle-aged and older adults, brain networks, UK Biobank

## Abstract

Mental health disorders are increasingly prevalent in middle-aged and older adults, a population undergoing substantial brain network reorganization. We aimed to identify whole-brain connectivity patterns associated with transdiagnostic psychiatric dimensions and their links to mental health trajectories and mortality. We analyzed resting-state functional connectivity from the UK Biobank (N = 6529) using multivariate partial least squares analysis to identify latent variables linking brain networks with mental health symptoms. Associations with longitudinal mental health outcomes and mortality risk were examined. Validation of the psychopathology-linked connectome constructs was conducted in HCP-Aging (N = 697) and a Singapore-based community dwelling elderly cohort known as the SG70 Study (N = 943). Two robust latent variables emerged. The first represented a general psychopathology factor (p = 0.0006, 28.0% of the overall covariance), marked by altered connectivity in the somatomotor and default mode networks. The second (p < 0.0001, 17.2% of the overall covariance) distinguished affective disorders from alcohol use disorder via attentional and subcortical network patterns. Importantly, the general psychopathology brain scores differentiated groups with varying future depression trajectories (F(3) = 16.47, p < 0.0001) and were linked to elevated mortality risk (HR = 1.31, CI [1.06–1.62], p = 0.014). This same connectivity signature was also associated with general mental health outcomes in HCP-Aging (rho = 0.13, p = 0.015) and depression in SG70 (rho = 0.07, p = 0.031), demonstrating cross-country and multiethnic robustness. Our findings reveal a stable, interpretable brain connectome-based signature of general psychopathology in later life. This work provides insight into mechanisms of vulnerability and suggests that brain-based markers may help indicate risk and differentiate patterns of symptom persistence, transition, and remission across disorders in aging populations.

## 1 Introduction

Mental health problems in middle-aged adults represent a major public health concern, markedly reducing life satisfaction and well-being (Wilk et al., 2024). Yet, despite growing attention to youth and late-life mental health, midlife, typically ages 40–60 years, remains underrepresented in research and policy (Lachman, 2004). Integrating midlife populations into lifespan and aging studies is crucial as midlife offers an opportunity for early detection and intervention that may prevent cognitive and functional decline in later life (Antal et al., 2025; Chan et al., 2018; Hildon et al., 2010; Mahdipour et al., 2015).

Neuroimaging offers a promising avenue for elucidating the neurobiological substrates of mental disorders, grounded in the view that these disorders represent disruptions of large-scale brain systems (Insel & Quirion, 2005). Convergent evidence indicates shared alterations across psychiatric disorders, particularly within the salience, executive control, and default mode networks—the core “triple network” model (Menon, 2011, 2019). At the same time, disorder-specific deviations have also been observed, aligning with the hierarchical model of psychopathology (Forbes et al., 2021; Wright et al., 2013). This framework not only accounts for shared neurobiological features but also provides a mechanistic explanation for the high prevalence of comorbidities across psychiatric diagnoses (Kessler, 1997; Kroenke & Price, 1993), making it a compelling model for studying macroscopic brain dysfunction. However, conventional univariate analyses are limited in capturing the distributed and interdependent brain–behavior patterns underlying complex psychiatric phenotypes (Lessov-Schlaggar et al., 2016). Given the dimensional and transdiagnostic nature of psychopathology (Forbes et al., 2021), multivariate approaches are needed to disentangle overlapping and disorder-specific neural signatures.

Multivariate neuroimaging studies in youth have unveiled a hierarchical organization in brain connectivity (Lees et al., 2020; Xia et al., 2018), revealing both shared and unique neural patterns across distinct disorders, with the default, control, and attentional networks as the most affected across all dimensions. However, research investigating the neural architecture of psychopathology in adults, particularly middle-aged and older individuals, remains limited, often constrained by small sample sizes and cross-sectional designs (Horga et al., 2014; Schnack & Kahn, 2016; Szucs & Ioannidis, 2020). Given the dynamic reorganization of brain functional networks during mid-to-late adulthood (Naik et al., 2017), how the transdiagnostic hierarchy of psychopathology manifests in this context is not yet well understood. This calls for large-scale, longitudinal, and transdiagnostic studies to capture the evolving and multidimensional nature of psychopathology in aging.

This study endeavors to address these gaps by investigating brain functional phenotypes in middle-aged and older adults from the UK Biobank cohort (Sudlow et al., 2015), a richly characterized cohort with whole-brain imaging, behavioral assessments, and long-term follow-up in over 40,000 participants. This dataset enables a systematic, transdiagnostic examination of mental health dimensions in a real-world aging population, including those with subthreshold psychiatric symptoms who are typically underrepresented in clinical studies. Using partial least squares (PLS), we investigated how variation in functional connectivity relates to latent dimensions of psychopathology, independent of diagnostic labels. This method allows us to consider comorbid symptoms and capture shared and unique brain–behavior associations across the spectrum of mental health. We hypothesize a general psychopathology factor spanning multiple symptom domains alongside distinct profiles associated with specific conditions. Importantly, we hypothesize that psychopathological manifestations might not only be seen within high-order networks, but also in the sensory networks, building upon previous findings in younger adult multivariate studies (Kebets et al., 2019). We further hypothesize that the identified brain network phenotypes will provide deeper insights into the neural substrates underlying vulnerability to predict future mental health trajectory and mortality outcomes. Lastly, we also seek to validate the identified brain phenotypes in independent cohort across different ethnicities.

## 2 Methods

### 2.1 Participants

Data were obtained from the UK Biobank (application 25163), with ethical approval from the National Research Ethics Service Committee North West - Haydock (11/NW/0382) (Alfaro-Almagro et al., 2018; Sudlow et al., 2015). We excluded participants with any reported cancer, non-cancer, and stroke up until the first imaging visit (details in Supplement 1, Supplementary Table S1). We only included participants who completed the online mental health questionnaire and had brain imaging data. After quality control (Supplement 1), 6,529 scans were retained. Demographic and behavioral characteristics are summarized in Table 1.

**Table 1.** Participant characteristics.

	Characteristics	Total (n = 6529)
Demographics	Sex, male:female	2814:3715
	Age at baseline scan, years, mean (SD)	62.90 (7.49)
	Age finishing MHQ, years, mean (SD)	61.75 (7.34)
	Site (Cheadle:Reading:Newcastle:Bristol)	3933:981:1608:7
MRI	Head size, mean (SD)	1.30 (0.12)
	Mean relative motion, mm, mean (SD)	0.12 (0.05)
	Time since first acquisition, years, mean (SD)	3.56 (1.58)
MHQ measures	AUDIT	6.77 (4.86)
	GAD-7	3.13 (3.92)
	PHQ-9	3.81 (4.26)
	PCL-6	10.09 (3.29)
	Psychotic events	0.18 (0.83)

Note: AUDIT: Alcohol Use Disorders Identification Test, GAD7: General Anxiety Disorder-7 questions, MHQ: mental health questionnaire, PHQ9: Patient Health Questionnaire 9-question version, PCL6: post-traumatic stress disorder Check List – civilian short version.

### 2.2 Mental health questionnaire

The mental health questionnaire collected in 2016 was utilized in this study (Davis et al., 2020). We chose 36 items from established questionnaires capable of reflecting not only the presence but also the severity of different disorders, namely the Alcohol Use Disorders Identification Test (AUDIT) (Pradhan et al., 2012), Generalised Anxiety Disorder Questionnaire 7-question (GAD-7) (Kroenke et al., 2010), Patient Health Questionnaire 9-question (PHQ-9) (Kroenke et al., 2010), post-traumatic stress disorder Check List – civilian short version (PCL-6) (Lang & Stein, 2005), and number of psychotic-like experiences (Nuevo et al., 2012) (full list in Supplementary Table S2). Items were log-transformed prior to analysis.

### 2.3 Image acquisition and preprocessing

On top of the standard UK Biobank preprocessing (Alfaro-Almagro et al., 2018), we applied additional steps following a prior study (Chong et al., 2017) to the resting-state functional magnetic resonance imaging (fMRI) data. Specifically, we extracted time series for 400 cortical regions of interest (ROIs) (Schaefer et al., 2018) and 30 subcortical ROIs (Choi et al., 2012; Tzourio-Mazoyer et al., 2002), organized into 9 networks: control, default mode, dorsal attention, limbic, salience/ventral attention, somatomotor, temporal, visual, and subcortical networks (Yeo et al., 2011). In total, 10 ROIs were excluded due to a lack of coverage, leaving 420 ROIs for analyses. Static functional connectivity (FC) matrices were calculated using Pearson’s correlation, Fisher z-transformed and demeaned before further analyses.

### 2.4 Partial least squares correlation

To identify the relationship between whole-brain functional connectivity and multiple mental health outcomes, we first regressed the effects of the “simple” set of confounders (excluding task fMRI motion) (Alfaro-Almagro et al., 2021) (Supplement 1) out of the FC matrices and the mental health item scores. We then submitted the residuals to a partial least squares (PLS) correlation analysis (Krishnan et al., 2011), extracting latent variables (LVs) via singular value decomposition. Significance was assessed with 5,000 permutations. Each LV captured patterns of brain and disorder salience, reflecting their correlation within the domain (Krishnan et al., 2011). Each participant was characterized by a brain score and an impairment score, reflecting their expression of specific brain networks and impairment profiles. Correlations between brain scores and each questionnaire item were calculated with bootstrapping derived standard error (1000 iterations) (Krishnan et al., 2011). Brain scores were then standardized prior to any future outcome analyses (1 unit = 1 SD) to ensure interpretability.

For gauging the contribution of individual brain networks to the relevant dimensional phenotypes, we derived the absolute values of the brain salience across all connections for each ROI belonging to the respective network to estimate their importance to the respective LV. A permutation test (1000 iterations) was employed to evaluate the significance of this important measure for each brain network by randomly shuffling the derived values across the brain.

In order to validate the internal consistency of our PLS results, we randomly split the dataset in half 100 times and repeated the PLS (Nakua et al., 2024) (Supplement 1).

### 2.5 Brain connectivity patterns and clinical trajectories

To further study the clinical relevance of the derived brain scores, we assessed the relationship between the standardized brain scores from the first two LVs and future outcomes. A subset of participants underwent a second round of mental health questionnaire with AUDIT (n = 4487), GAD-7 (n = 5462), and PHQ-9 (n = 5460).

Participants were categorized based on persistence or change in condition using a cutoff of 8 was used for AUDIT to identify hazardous drinking (Skipsey et al., 1997) and a cutoff of 10 for both GAD-7 and PHQ-9 to determine current anxiety and depression (Kroenke et al., 2010). A series of ANOVAs were carried out to compare the brain scores across groups, with Tukey’s post hoc tests and FDR correction.

To further evaluate whether baseline brain scores predicted future psychiatric symptom severity independent of baseline symptom burden, we also fitted linear regression models with baseline symptom severity as a covariate (Supplement 1).

### 2.6 Prediction of mortality risk

To evaluate the value of the brain phenotypes in predicting mortality risk, we assessed the relationship between the standardized brain scores and mortality. Mortality was confirmed in a subset of 6528 individuals, with 100 determined to be deceased by the censoring date of November 30, 2022. Cox proportional hazards regression was employed to estimate the mortality risk associated with the standardized brain scores obtained from the first two LVs after accounting for age, sex, years of education, and social economic status as measured by the Townsend Deprivation Index (Rask-Andersen et al., 2021). Effect sizes were reported as hazard ratios (HRs), interpreted as the relative change in mortality risk per 1 standard deviation increase in brain score. Model performance was compared against a covariate-only baseline model using a likelihood ratio test.

### 2.7 Cross-cohort validation of the general psychopathology factor

In order to assess the generalizability and specificity of our identified brain phenotypes, we projected our FC findings in the HCP-Aging dataset (Bookheimer et al., 2019), with enrollment, protocols, and preprocessing described previously (Glasser et al., 2013; Harms et al., 2018). We included 376 participants between the age of 18 and 59 years (47.85 ± 7.02 years, 160 males) with no missing behavioral and fMRI data.

As a measure of general mental health in HCP-Aging, we utilized the total problem score, which is the sum of the items in the Achenbach Adults Self-Report questionnaire (ASR) (Achenbach & Rescorla, 2003) and controlled for age, age squared, sex, age * sex before further analyses.

We extracted the resting-state time series and derived the FC matrix using the previously described procedure in the UK Biobank analysis. We demeaned and residualized FC matrices for age, age squared, sex, age * sex, and motion before deriving the brain scores. For each participant, we multiplied the residualized FC matrix with the brain salience for each of the two LVs, took the sum of the upper triangular values, and normalize them to derive individual brain scores. We then correlated these scores with the residualized total problem scores using Spearman’s correlation.

As an additional validation, we also looked at the sum of the items from the Achenbach Older Adult Self-Report (OASR) for participants older than 60 years and carried out a similar analysis (Supplement 1).

### 2.8 Cross-ancestry validation of the general psychopathology factor in an Asian cohort

To assess the cross-ethnic generalizability of our identified brain phenotypes, we projected the UK Biobank-derived FC findings onto participants from the SG70 study, which is nested within the Singapore Chinese Health Study (SCHS) (Qin et al., 2025). This study recruited participants aged 68–80 years during the period of September 2021 to March 2025. In total we included 943 participants (74.9 ± 2.58 years, 441 males) with no missing behavioral and fMRI data.

fMRI acquisition and preprocessing details are given in Supplement 1. Resting-state fMRI data were again preprocessed following the same procedures. We demeaned and residualized FC matrices for age, sex, age * sex, and motion before deriving the brain scores.

As a measure of mental health in this cohort, we utilized the Geriatric Depression Scale - 15 items (GDS) (Burke et al., 1991). For continuous analyses, we correlated individual brain scores with the GDS total scores using Spearman’s correlation while controlling for age, sex, age * sex. In order to differentiate participants who met criteria for depressive disorders with GDS score greater than 5 (Burke et al., 1991), we fitted logistic regression models to predict depressive status using brain scores, adjusting for the same covariates.

## 3 Results

### 3.1 Functional connectivity relates to dimensions of psychopathology

The PLS analysis yielded six statistically significant latent variables (LVs) (p < 0.05, Supplementary Fig. S1), namely latent variables 1, 2, 3, 6, 8, 10, as ranked by the amount of covariance explained. We only focused on the top two LVs as they explained almost half of the total covariance, with the remaining latent variables all explaining less than 10% of the total covariance each.

**Fig. 1.**
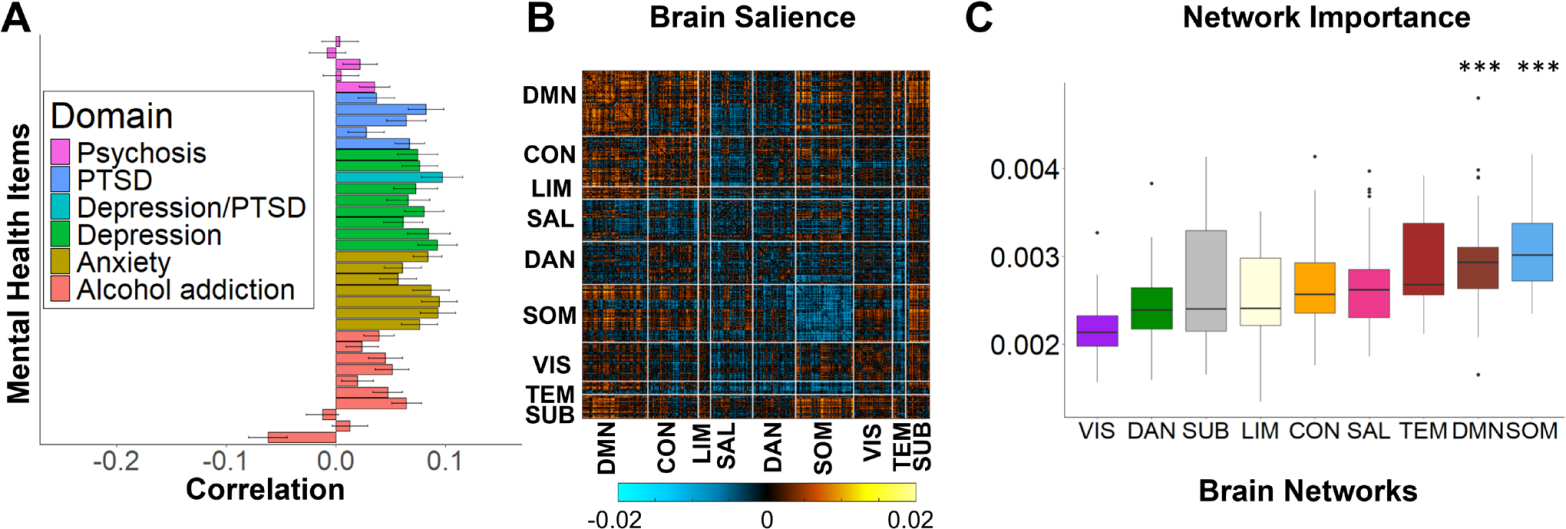
Brain functional connectome signature underlies a general psychopathology factor. The first latent variable of partial least squares analysis reflects the relationship between brain functional connectivity and general psychopathology. (A) Correlations with bootstrapped standard error of outcome items and brain scores. (B) Brain salience reflecting the contribution of each connection. (C) Important values reflected relative contribution of functional networks, highlighting the contribution of somatomotor and default mode networks. PTSD: posttraumatic stress disorder, DMN: default mode, CON: control, LIM: limbic, SAL: salience/ventral attention, DAN: dorsal attention, SOM: somatomotor, VIS: visual, TEM: temporal, SUB: subcortical. ***p ≤ 0.001.

The first LV, explaining 28.00% of the overall covariance (p = 0.0006), represented a general psychopathological factor, where most of the mental health items showed positive correlations to the brain scores, except for one notable alcohol-related item that measured drinking frequency (Fig. 1A). Therefore, we will refer to this LV as the general psychopathology factor from henceforth. Looking at the brain salience profile, individuals with higher psychiatric burden exhibited lower functional connectivity within the somatomotor network, higher functional connectivity within the default mode network, and between default mode and control network, as well as higher connectivity between the somatomotor network and default, control, and salience/ventral attention networks (Fig. 1B). The most important network underlying this latent variable was the somatomotor network followed by the default mode network (Fig. 1C). This finding suggested that an overall psychopathology factor may be underscored by primarily aberrant connectivity within and between somatomotor and default mode networks.

**Fig. 2.**
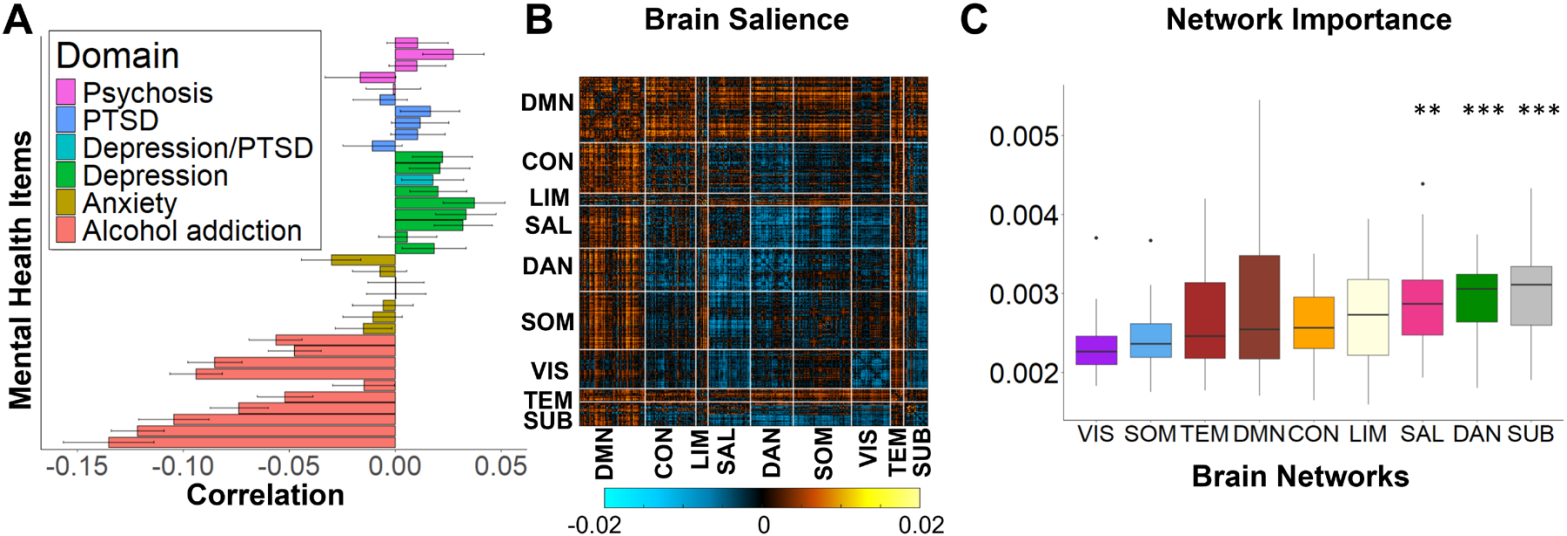
Functional connectome signature reveals the divergence between alcohol use disorder and depression/PTSD. The second latent variable of the partial least squares analysis delineates how brain functional connectivity differentiates alcohol use disorder from depression/PTSD. (A) Correlations with bootstrapped standard error of outcome items and brain scores. (B) Brain salience reflecting the contribution of each connection. (C) Important values reflected relative contribution of functional networks, highlighting the contribution of subcortical and attentional networks. PTSD: posttraumatic stress disorder, DMN: default mode, CON: control, LIM: limbic, SAL: salience/ventral attention, DAN: dorsal attention, SOM: somatomotor, VIS: visual, TEM: temporal, SUB: subcortical. **p ≤ 0.01, ***p ≤ 0.001.

The second LV, explaining 17.16% of the overall covariance (p < 0.0001), emphasized a distinction between alcohol use disorder and symptoms related to depression and PTSD, with strong positive correlations for alcohol-related items and negative correlations for most depression/PTSD-related syndromes (Fig. 2A). Therefore, we will refer to this LV as the addiction vs. affective factor from henceforth. Looking at the brain salience profile, we noted that lower alcohol use disorder, but worse problems related to depression and PTSD, was associated with lower functional connectivity in subcortical regions and between the dorsal and ventral attention networks (Fig. 2B). The most important network in this latent variable was the subcortical network followed by the dorsal attention and salience/ventral attention networks (Fig. 2C). These findings suggest that divergent patterns of psychopathology may be underpinned by distinct disruptions in subcortical and attentional network connectivity.

We also observed similar results in the split half analysis (Supplement 1, Supplementary Table S3, Supplementary Fig. S2, Supplementary Fig. S3, Supplementary Fig. S4), with a latent variable representing general psychopathology and another one separating different disorders supported by a similar set of networks as previously detailed.

### 3.2 Brain network phenotypes relate to longitudinal mental health trajectories

**Fig. 3.**
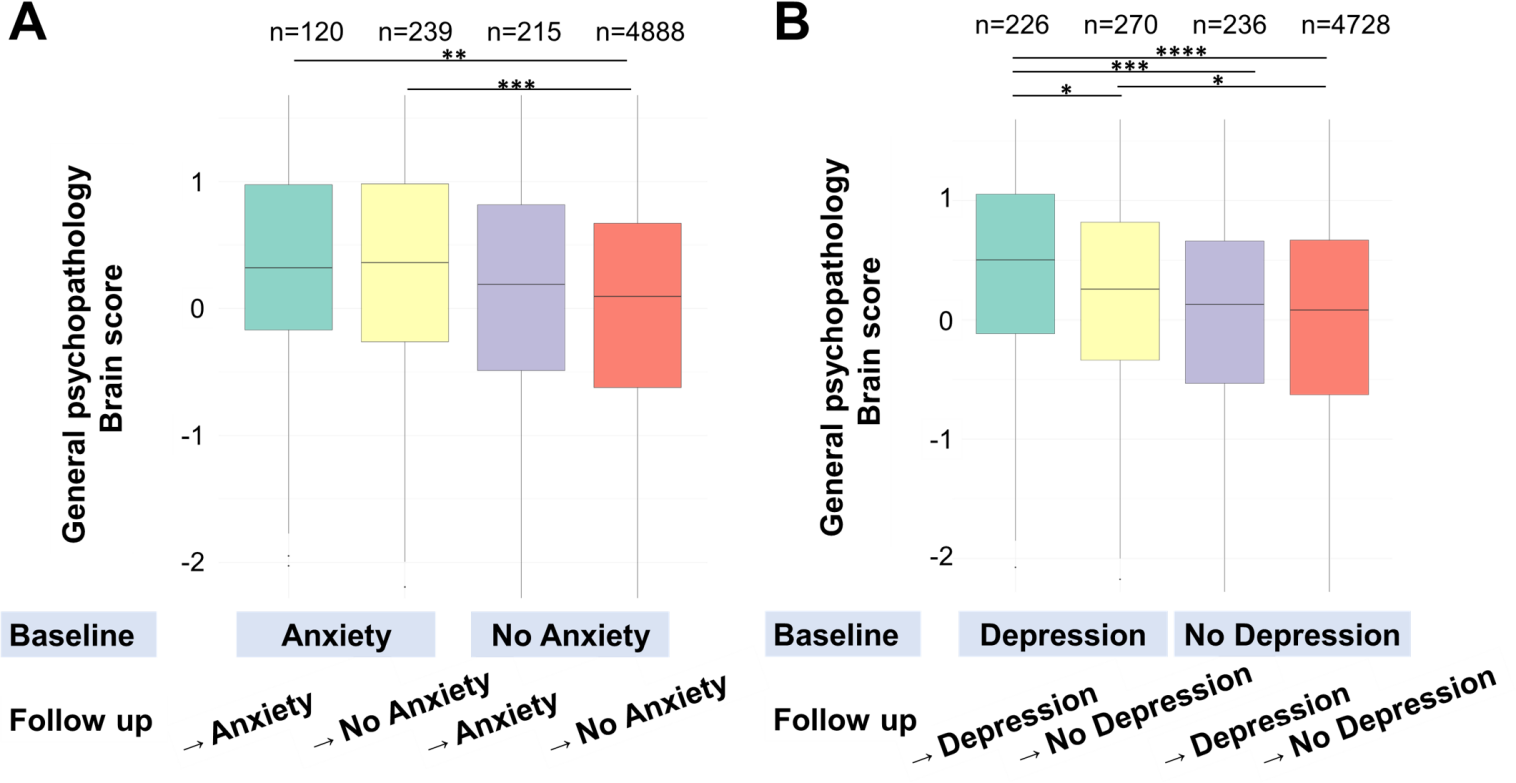
Brain scores might predict transition and remission across different mental health disorders. (A) General psychopathology brain score plotted for four groups with different current and future anxiety status. (B) General psychopathology brain score plotted for four groups with different current and future depression status. Box plots displayed the median and the interquartile range (IQR). The whisker extended from the box to the largest/smallest value no farther than 1.5∗ IQR. All values beyond this range were plotted individually. *p ≤ 0.05, **p ≤ 0.01, ***p ≤ 0.001, ****p ≤ 0.0001.

Looking at future outcomes, we found that there were significant differences in baseline general psychopathology brain scores across the four clinical trajectories for each of the three mental conditions, namely anxiety, depression, and hazardous drinking (Fig. 3, Supplementary Table S4). Meanwhile for the addiction vs. affective factor, the group differences were only seen in hazardous drinking (Supplementary Table S4).

Post hoc analyses revealed that for anxiety, general psychopathology brain scores differentiated between the group who remained anxiety-free over time (in red) versus the group with current anxiety regardless of their future outcomes (green and yellow) (Fig. 3A, Supplementary Table S5). We saw similar findings for depression, where those with no depression at baseline (in red) had a lower score than those with current depression (green and yellow) (Fig. 3B, Supplementary Table S5). Importantly, groups with persistent depression (in green) also showed higher general psychopathology brain scores than those who only developed depression in the future (in purple) and those who remitted (in yellow) (Fig. 3B, Supplementary Table S5). We observed similar findings with reduced significance after controlling for baseline depression (see Supplement 1, Supplementary Table S7, Supplementary Table S8).

Lastly, groups who only developed drinking problems in the second time point showed lower general psychopathology brain scores than all the other three groups (Supplementary Fig. S5, Supplementary Table S5), while groups with no drinking problems throughout showed higher addiction vs. affective factor scores than groups with baseline drinking problems (Supplementary Fig. S5, Supplementary Table S6). The latter relationship remained after controlling for baseline symptom burden (see Supplement 1, Supplementary Table S7, Supplementary Table S8). This suggested that brain scores might be able to indicate risks and vulnerabilities by differentiating transition and remission across different disorders.

### 3.3 General psychopathology brain network scores at baseline predict mortality risk

**Fig. 4.**
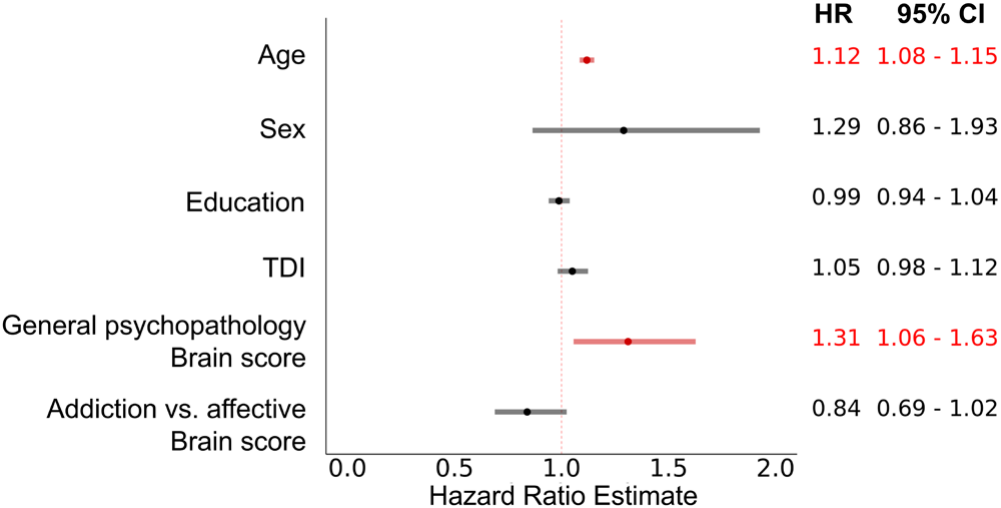
Higher general psychopathology-associated brain score led to higher mortality rate. Forest plot showing hazard ratio estimate and 95% confidence interval. Variables with significant effects, including age and general psychopathology brain score, are highlighted in red. CI: confidence interval, HR: hazard ratio, TDI: Townsend deprivation index.

The Cox model revealed that the general psychopathology brain score but not the addiction vs. affective brain score (Fig. 4) was a significant predictor of mortality (hazard ratio (HR) 1.31, 95% confidence interval (CI) [1.06–1.62], p = 0.014), even after accounting for chronological age, sex, years of education, and social economic status (concordance = 0.74, loglikelihood = -839.45*)*. This indicates that for every 1 SD increase in the standardized brain score, the odds of mortality increase by 31%. Age was another significant predictor of mortality (HR 1.12, CI [1.08–1.15], p = 5.21*10^-13^). This model significantly outperformed the base model without brain scores (concordance = 0.73, loglikelihood = -844.26, χ^2^ = 9.6234, p = 0.0081). These findings support the potential value of general psychopathology associated brain phenotype as a prognostic marker of mortality in this context. .

### 3.4 General psychopathology connectome-based construct predicts mental health in HCP-aging

In HCP-Aging, as expected, the projected brain scores corresponding to the general psychopathology factor showed a significant positive correlation with total problem scores (rho = 0.12, p = 0.015), indicating that higher expression of this neural phenotype was associated with greater psychopathology burden. In contrast, brain scores corresponding to the addiction-versus-affective factor were not associated with this score (rho = -0.07, p = 0.16), highlighting its specificity. Similar patterns were observed when using the Older Adult Self-Report (OASR) in older HCP-Aging sample (Supplement 1). These findings support the stability of the general psychopathology-related brain phenotype across independent cohorts and across countries.

### 3.5 General psychopathology connectome-based construct predicts depression in an Asian population cohort

Within the Singaporean SG70 cohort, brain scores corresponding to the general psychopathology factor showed a significant positive association with GDS total scores (rho = 0.07, p = 0.031), indicating that higher expression of this phenotype was associated with greater depressive symptom burden. In contrast, brain scores corresponding to the addiction-versus-affective factor were not significantly correlated with GDS total scores (rho = -0.03, p = 0.43), again highlighting the robustness and domain specificity of the brain phenotypes.

Consistent patterns were observed in logistic regression analyses predicting clinically relevant depressive symptoms. Only the general psychopathology scores significantly predicted depressive status (odds ratio = 1.36, 95% CI = 1.00–1.84, p = 0.049). This indicates that for every 1 SD increase in the standardized brain score, the odds of depression increase by 35%. The other brain score did not show a significant association with depressive status (odds ratio = 0.89, 95% CI = 0.67–1.17, p = 0.39).

These findings demonstrate that the general psychopathology-related brain phenotype generalizes across populations and ethnicities, extending from the primarily Caucasian UK Biobank cohort to the Asian population. This finding also supports the contribution of this brain phenotype in relation to depression, mirroring our main findings within UK Biobank.

## 4 Discussion

In this study, we examined how functional brain connectivity relates to psychopathology in middle-aged and older adults, an underrepresented group in neuroimaging research. Using a large-scale, population-based cohort with longitudinal follow-up, we identified two LVs linking brain network phenotypes to mental health symptoms. The first LV captured a general psychopathology factor, marked by altered connectivity in the somatomotor and default mode networks, robustly associated with symptom burden across independent datasets. The second LV differentiated affective symptoms from alcohol use disorder, with opposing connectivity patterns in subcortical and attentional networks. Higher general psychopathology brain scores predicted persistent depression over time and increased mortality risk, highlighting their potential as transdiagnostic markers of long-term vulnerability.

The identification of multiple significant LVs underscores the complexity of the relationship between functional connectivity and psychopathology in a population cohort. Of particular interest were the first and second LVs, which explained a substantial portion of the overall covariance. The first LV represented a general psychopathological factor, whereas the second highlighted distinctions between alcohol use disorder and symptoms related to depression and PTSD. Functional connectivity patterns thus capture underlying psychopathology dimensions beyond traditional diagnostic categories, following the hierarchical model (Forbes et al., 2021).

We noted individuals with higher psychiatric burden exhibited functional connectivity aberrations within the somatomotor network and default mode network, as well as between-network connectivity somatomotor, default mode, control network, and salience networks, consistent with previous findings (Doucet et al., 2020; Kebets et al., 2019; Menon, 2011, 2019; Sha et al., 2019; Zhang et al., 2024). Permutation analysis highlighted the important role of somatomotor and default mode networks, suggesting dysregulation in sensorimotor processing (Penfield & Boldrey, 1937) and self-referential processing (Davey et al., 2016). These networks have been previously shown to be implicated in transdiagnostic dimensions in younger patients (Doucet et al., 2020; Kebets et al., 2019; Sha et al., 2019), which suggested their pivotal and enduring role.

In the middle-aged group, people started experiencing notable decline in motor functioning (Hoogendam et al., 2014). Studies have shown that physical activities starting in middle-aged and even in older adulthood can improve cognitive ability and mental health (Gow et al., 2017; Smith & Merwin, 2021). However, the existing literature has mainly focused on looking at the effects of activities on higher-order networks involved in the triple network models (Menon, 2011; Smith & Merwin, 2021), with the most common being the default mode networks (Bray et al., 2021). However, these association networks failed to explain and mediate the relationship between midlife activities and later-life outcomes (Raykov et al., 2024), prompting a closer look at the somatomotor network. Moving away from motor functioning and physical activities, this network is also highly involved in social cognition through the sensorimotor and informational coupling (Engel et al., 2022). The interaction between the somatomotor and default mode network plays a crucial role in processing and perceiving external stimuli to generate a social behavior (Love et al., 2015; Spreng & Andrews-Hanna, 2015). Social cognition has been shown to be impaired in various psychiatric disorders (Erdeniz et al., 2017; Gallagher & Varga, 2015; Senju, 2013; Weightman et al., 2014), which might explain the important role of the two abovementioned networks in the transdiagnostic dimension identified in this current work. Our findings, therefore, both reaffirm the importance of the default mode network and also emphasize the necessity for increased attention to the somatomotor region when discussing etiology, preventive strategies, and therapeutic interventions for psychopathological conditions.

Importantly, these observations also raise the possibility that the contribution of the somatomotor network to general psychopathology is not entirely fixed, but may be modulated by environmental and sociocultural context (Borsboom et al., 2019). Differences in physical activity, social interaction norms, and age-related motor decline may, therefore, influence the degree to which somatomotor dysfunction contributes to psychopathology (Asp et al., 2025; Buchman et al., 2010; Litwin, 2012; Park et al., 2024). This is consistent with evidence that sensory–association (S-A) axis organization may vary across populations due to environmental inputs (Han et al., 2025; Sydnor et al., 2023).

While the first LV looked at the shared etiology, the second LV highlighted aberrant connectivity linked to subcortical and attentional networks, reflecting the diverging patterns of functional connectivity corresponding to alcohol use disorder versus depression and PTSD. This divergence might reflect the well-described externalizing–internalizing latent constructs (Gustavson et al., 2020). Externalizing disorders, exemplified here by alcohol use disorder, typically manifest as outward behaviors such as impulsivity, risk taking, and inattention (Samek & Hicks, 2014). These behaviors are often linked to dysfunctions in reward processing and impulse control regions, specifically the salience and subcortical networks (Baselmans et al., 2022; Tolomeo & Yu, 2022; Zhu et al., 2017), as well as the attentional networks via their regulation of distinct attentional processes (Baselmans et al., 2022; Song et al., 2020; Vossel et al., 2014; Zehra et al., 2019). However, the internalizing disorders, which include both depressive and traumatic disorders (American Psychiatric Association & Association, 2013), are often characterized by high levels of internal distress (Tandon et al., 2009). This “intropunitive” manifestation, while standing in stark contrast of the outward behaviors associated with externalizing disorders, has been shown to involve alterations in similar pathways involving altered attention (Chen et al., 2019; Hostinar & Cicchetti, 2020; Pollak, 2015; Russman Block et al., 2020; Shields & Cicchetti, 1998) as well as blunted reward response (Brown et al., 2014; Hostinar & Cicchetti, 2020; Zeng et al., 2012). Our results, therefore, demonstrated the delicate balance that needs to be maintained for a healthy mental state, where disruptions to the same system can lead to distinct disease outcomes.

We noted that brain scores could help differentiate between groups with differing baseline and future outcomes. Importantly, groups with persistent depression also showed higher general psychopathology brain scores than those who only developed depression in the future and those who remitted. This suggests that brain network phenotypes may reflect differences among subgroups with varying clinical trajectories, though these associations should be interpreted cautiously given the likely influence of baseline symptom levels and temporal autocorrelation. Future work should directly evaluate whether these brain-derived markers provide incremental predictive value beyond baseline clinical severity in externally validated cohorts, particularly in more clinically enriched samples. In the present study, the relatively healthy population-based sample likely limits the range of symptom severity and trajectory divergence, which may in turn constrain detectable brain–behavior associations. Studies with longer follow-up intervals and higher clinical burden will be important to determine whether these latent dimensions meaningfully improve individual-level prediction or stratification beyond established clinical predictors. Importantly, beyond associations with psychiatric trajectories, the general psychopathology brain scores were also highlighted as a significant predictor of mortality, even after accounting for chronological age, sex, years of education, and social economic status. All in all, this supports the role of this connectome construct as markers of vulnerability.

The consistency of our findings across analyses, including split-half validation and external dataset projection, strengthens the reliability of our results. In HCP-Aging, a US-based cohort, total problem scores were predicted only by the general psychopathology-related brain scores, and not by the LV capturing distinctions between disorders. Extending these observations, in the older Asian SG70 cohort, again only the general psychopathology construct was associated with depressive symptoms, whereas the addiction vs. affective factor was not, confirming its domain specificity. Notably, the effect sizes observed in the external validation cohorts were more modest overall than the discovery sample. This reduction in magnitude may be expected in independent validation settings and could reflect several factors, including potential overfitting in the primary cohort and imperfect alignment of behavioral constructs across datasets (e.g., differences in depression phenotyping in SG70) (Rosenblatt et al., 2024; Shafiei et al., 2025). In addition, the effect size observed in SG70 was attenuated relative to HCP-Aging, which may in part reflect differences in cohort characteristics, particularly the older age distribution in SG70, as well as potential population-specific differences in normative brain aging trajectories. Emerging evidence suggests that brain developmental and aging milestones may vary across populations (Sun et al., 2025; Zhuo et al., 2026), which could modulate the expression of brain–behavior associations in late life. Taken together, these results indicate that the general psychopathology-related brain phenotype is robust across cohorts and broadly observable across different ethnic backgrounds, while also highlighting that its effect magnitude may be influenced by differences in measurement, as well as population-specific demographic composition and biological aging processes.

From a translational perspective, these findings also highlight the importance of considering population specificity in brain-based prediction models. Future work could benefit from integrating such transdiagnostic neural signatures with population-specific normative brain references, as models trained in non-representative samples may not generalize optimally across diverse groups. Developing region- and ancestry-informed “brain charts” may, therefore, help improve the fairness and accuracy of individual-level risk stratification in clinical applications (Han et al., 2025; Sun et al., 2025; Zhuo et al., 2026).

This study focused on a largely healthy population due to the nature of the imaging subsample within the UK Biobank dataset (Lyall et al., 2022). Therefore, the psychopathology-related imaging phenotypes identified in this paper should mainly be interpreted in terms of variations of psychological well-being, risks, and vulnerabilities. Despite that, the fact that we identified a transdiagnostic dimension underscored by somatomotor network, replicating a healthy control–patient cohort study (Kebets et al., 2019), indicated that these neuroimaging signatures persist upon disease manifestation.

Despite the strengths of our study, we shared many limitations with previous mental health studies using UK Biobank (Roelfs et al., 2023) due to a predominantly White and British population (Fry et al., 2017; Sudlow et al., 2015) and reliance on self-reported questionnaires (Davis et al., 2020; Gove & Geerken, 1977). In addition, the absence of alcohol misuse data in external cohorts prevented full cross-dataset validation of the second latent variable, limiting evaluation of its cross-cultural generalizability. This reflects a broader challenge in psychiatric research, where differences in phenotype harmonization can constrain direct comparability across studies (Shafiei et al., 2025). Nonetheless, the first latent variable showed strong robustness and replicability across cohorts, supporting their relevance to transdiagnostic psychopathology.

In conclusion, our study provides compelling evidence for the association between functional connectivity patterns and psychopathology, as well as the predictive power of baseline brain scores for future mental health outcomes and mortality risk. Importantly, these findings were robust across multiple cohorts and ethnically diverse populations. All in all, this underscores the significance of comprehending brain network phenotypes related to mental disorders within the framework of transdiagnostic dimensions. By elucidating the neurobiological underpinnings of mental health disorders, our findings have important implications for advancing our understanding of psychiatric pathophysiology and informing personalized approaches to mental healthcare.

## Supplementary Material

Supplementary Material

## Data Availability

The data used in this study were obtained from the UK Biobank and the Human Connectome Project in Aging (HCP-Aging). These data are available to qualified researchers through application to the respective data access procedures. All code used in these analyses is publicly available on GitHub at https://github.com/hzlab/2026_Nguyen_ImagingNeuroscience_Connectome_Psychopathology

## References

[IMAG.a.1308-b1] Achenbach, T. M., & Rescorla, L. (2003). Manual for the ASEBA adult forms & profiles. Burlington, VT: University of Vermont, Research Center for Children, Youth and Familes. https://aseba.org/wp-content/uploads/2019/01/ASEBA-Reliability-and-Validity-Adult.pdf

[IMAG.a.1308-b2] Alfaro-Almagro, F., Jenkinson, M., Bangerter, N. K., Andersson, J. L. R., Griffanti, L., Douaud, G., Sotiropoulos, S. N., Jbabdi, S., Hernandez-Fernandez, M., Vallee, E., Vidaurre, D., Webster, M., McCarthy, P., Rorden, C., Daducci, A., Alexander, D. C., Zhang, H., Dragonu, I., Matthews, P. M.,… Smith, S. M. (2018). Image processing and quality control for the first 10,000 brain imaging datasets from UK Biobank. Neuroimage, 166, 400–424. 10.1016/j.neuroimage.2017.10.034 29079522 PMC5770339

[IMAG.a.1308-b3] Alfaro-Almagro, F., McCarthy, P., Afyouni, S., Andersson, J. L. R., Bastiani, M., Miller, K. L., Nichols, T. E., & Smith, S. M. (2021). Confound modelling in UK Biobank brain imaging. NeuroImage, 224, 117002. 10.1016/j.neuroimage.2020.117002 32502668 PMC7610719

[IMAG.a.1308-b4] American Psychiatric Association, D., & Association, A. P. (2013). Diagnostic and statistical manual of mental disorders: DSM-5 (Vol. 5). American Psychiatric Association Washington, DC. 10.1007/springerreference_179660

[IMAG.a.1308-b5] Antal, B. B., van Nieuwenhuizen, H., Chesebro, A. G., Strey, H. H., Jones, D. T., Clarke, K., Weistuch, C., Ratai, E.-M., Dill, K. A., & Mujica-Parodi, L. R. (2025). Brain aging shows nonlinear transitions, suggesting a midlife “critical window” for metabolic intervention. Proceedings of the National Academy of Sciences, 122(10), e2416433122. 10.1073/pnas.2416433122 PMC11912423 40030017

[IMAG.a.1308-b6] Asp, M., Simonsson, B., & Molarius, A. (2025). Physical activity, physical mobility, and mental health among persons 70 years or older: Results from a large population-based study in Sweden. Archives of Public Health, 83(1), 225. 10.1186/s13690-025-01718-w 40926247 PMC12418685

[IMAG.a.1308-b7] Baselmans, B., Hammerschlag, A. R., Noordijk, S., Ip, H., van der Zee, M., de Geus, E., Abdellaoui, A., Treur, J. L., & van ’t Ent, D. (2022). The genetic and neural substrates of externalizing behavior. Biological Psychiatry Global Open Science, 2(4), 389–399. 10.1016/j.bpsgos.2021.09.007 36324656 PMC9616240

[IMAG.a.1308-b8] Bookheimer, S. Y., Salat, D. H., Terpstra, M., Ances, B. M., Barch, D. M., Buckner, R. L., Burgess, G. C., Curtiss, S. W., Diaz-Santos, M., Elam, J. S., Fischl, B., Greve, D. N., Hagy, H. A., Harms, M. P., Hatch, O. M., Hedden, T., Hodge, C., Japardi, K. C., Kuhn, T. P.,… Yacoub, E. (2019). The lifespan human connectome project in aging: An overview. Neuroimage, 185, 335–348. 10.1016/j.neuroimage.2018.10.009 30332613 PMC6649668

[IMAG.a.1308-b9] Borsboom, D., Cramer, A. O. J., & Kalis, A. (2019). Brain disorders? Not really: Why network structures block reductionism in psychopathology research. Behavioral and Brain Sciences, 42, e2. 10.1017/S0140525X17002266 29361992

[IMAG.a.1308-b10] Bray, N. W., Pieruccini-Faria, F., Bartha, R., Doherty, T. J., Nagamatsu, L. S., & Montero-Odasso, M. (2021). The effect of physical exercise on functional brain network connectivity in older adults with and without cognitive impairment. A systematic review. Mechanisms of Ageing and Development, 196, 111493. 10.1016/j.mad.2021.111493 33887281

[IMAG.a.1308-b11] Brown, V. M., LaBar, K. S., Haswell, C. C., Gold, A. L., Beall, S. K., Van Voorhees, E., Marx, C. E., Calhoun, P. S., Fairbank, J. A., Green, K. T., Tupler, L. A., Weiner, R. D., Beckham, J. C., Brancu, M., Hoerle, J. M., Pender, M., Kudler, H., Swinkels, C. M., Nieuwsma, J. A.,… Mid-Atlantic, M. W. (2014). Altered resting-state functional connectivity of basolateral and centromedial amygdala complexes in posttraumatic stress disorder. Neuropsychopharmacology, 39(2), 351–359. 10.1038/npp.2013.197 23929546 PMC3870774

[IMAG.a.1308-b12] Buchman, A. S., Boyle, P. A., Wilson, R. S., James, B. D., Leurgans, S. E., Arnold, S. E., & Bennett, D. A. (2010). Loneliness and the rate of motor decline in old age: The rush memory and aging project, a community-based cohort study. BMC Geriatrics, 10(1), 77. 10.1186/1471-2318-10-77 20969786 PMC2975650

[IMAG.a.1308-b13] Burke, W. J., Roccaforte, W. H., & Wengel, S. P. (1991). The short form of the geriatric depression scale: A comparison with the 30-item form. Journal of Geriatric Psychiatry and Neurology, 4(3), 173–178. 10.1177/089198879100400310 1953971

[IMAG.a.1308-b14] Chan, D., Shafto, M., Kievit, R., Matthews, F., Spink, M., Valenzuela, M., & Henson, R. N. (2018). Lifestyle activities in mid-life contribute to cognitive reserve in late-life, independent of education, occupation, and late-life activities. Neurobiology of Aging, 70, 180–183. 10.1016/j.neurobiolaging.2018.06.012 30025291 PMC6805221

[IMAG.a.1308-b15] Chen, H., Liu, K., Zhang, B., Zhang, J., Xue, X., Lin, Y., Zou, D., Chen, M., Kong, Y., Wen, G., Yan, J., & Deng, Y. (2019). More optimal but less regulated dorsal and ventral visual networks in patients with major depressive disorder. Journal of Psychiatric Research, *110*, 172–178. 10.1016/j.jpsychires.2019.01.005 30654314

[IMAG.a.1308-b16] Choi, E. Y., Yeo, B. T. T., & Buckner, R. L. (2012). The organization of the human striatum estimated by intrinsic functional connectivity. Journal of Neurophysiology, 108(8), 2242–2263. 10.1152/jn.00270.2012 22832566 PMC3545026

[IMAG.a.1308-b17] Chong, J. S. X., Liu, S., Loke, Y. M., Hilal, S., Ikram, M. K., Xu, X., Tan, B. Y., Venketasubramanian, N., Chen, C. L.-H., & Zhou, J. (2017). Influence of cerebrovascular disease on brain networks in prodromal and clinical Alzheimer’s disease. Brain, 140(11), 3012–3022. 10.1093/brain/awx224 29053778 PMC5841199

[IMAG.a.1308-b18] Davey, C. G., Pujol, J., & Harrison, B. J. (2016). Mapping the self in the brain’s default mode network. Neuroimage, 132, 390–397. 10.1016/j.neuroimage.2016.02.022 26892855

[IMAG.a.1308-b19] Davis, K. A. S., Coleman, J. R. I., Adams, M., Allen, N., Breen, G., Cullen, B., Dickens, C., Fox, E., Graham, N., Holliday, J., Howard, L. M., John, A., Lee, W., McCabe, R., McIntosh, A., Pearsall, R., Smith, D. J., Sudlow, C., Ward, J.,… Hotopf, M. (2020). Mental health in UK Biobank - development, implementation and results from an online questionnaire completed by 157 366 participants: A reanalysis. BJPsych Open, 6(2), e18. 10.1192/bjo.2019.100 32026800 PMC7176892

[IMAG.a.1308-b20] Doucet, G. E., Janiri, D., Howard, R., O’Brien, M., Andrews-Hanna, J. R., & Frangou, S. (2020). Transdiagnostic and disease-specific abnormalities in the default-mode network hubs in psychiatric disorders: A meta-analysis of resting-state functional imaging studies. European Psychiatry, 63(1), e57. 10.1192/j.eurpsy.2020.57 32466812 PMC7355168

[IMAG.a.1308-b21] Engel, A. K., Verschure, P., Kragic, D., Polani, D., Effenberg, A. O., & König, P. (2022). Editorial: Sensorimotor foundations of social cognition. Frontiers in Human Neuroscience, 16, 971133. 10.3389/fnhum.2022.971133 35874160 PMC9305330

[IMAG.a.1308-b22] Erdeniz, B., Serin, E., İbadi, Y., & Taş, C. (2017). Decreased functional connectivity in schizophrenia: The relationship between social functioning, social cognition and graph theoretical network measures. Psychiatry Research: Neuroimaging, 270, 22–31. 10.1016/j.pscychresns.2017.09.011 29017061

[IMAG.a.1308-b23] Forbes, M. K., Sunderland, M., Rapee, R. M., Batterham, P. J., Calear, A. L., Carragher, N., Ruggero, C., Zimmerman, M., Baillie, A. J., Lynch, S. J., Mewton, L., Slade, T., & Krueger, R. F. (2021). A detailed hierarchical model of psychopathology: From individual symptoms up to the general factor of psychopathology. Clinical Psychological Science, 9(2), 139–168. 10.1177/2167702620954799 33758691 PMC7983870

[IMAG.a.1308-b24] Fry, A., Littlejohns, T. J., Sudlow, C., Doherty, N., Adamska, L., Sprosen, T., Collins, R., & Allen, N. E. (2017). Comparison of sociodemographic and health-related characteristics of UK biobank participants with those of the general population. American Journal of Epidemiology, 186(9), 1026–1034. 10.1093/aje/kwx246 28641372 PMC5860371

[IMAG.a.1308-b25] Gallagher, S., & Varga, S. (2015). Social cognition and psychopathology: A critical overview. World Psychiatry, 14(1), 5–14. 10.1002/wps.20173 25655144 PMC4329883

[IMAG.a.1308-b26] Glasser, M. F., Sotiropoulos, S. N., Wilson, J. A., Coalson, T. S., Fischl, B., Andersson, J. L., Xu, J., Jbabdi, S., Webster, M., Polimeni, J. R., Van Essen, D. C., & Jenkinson, M. (2013). The minimal preprocessing pipelines for the Human Connectome Project. Neuroimage, 80, 105–124. 10.1016/j.neuroimage.2013.04.127 23668970 PMC3720813

[IMAG.a.1308-b27] Gove, W. R., & Geerken, M. R. (1977). Response bias in surveys of mental health: An empirical Investigation. American Journal of Sociology, 82(6), 1289–1317. 10.1086/226466 889001

[IMAG.a.1308-b28] Gow, A. J., Pattie, A., & Deary, I. J. (2017). Lifecourse activity participation from early, mid, and later adulthood as determinants of cognitive aging: The Lothian birth cohort 1921. The Journals of Gerontology: Series B, 72(1), 25–37. 10.1093/geronb/gbw124 PMC5156497 27974473

[IMAG.a.1308-b29] Gustavson, D. E., Franz, C. E., Panizzon, M. S., Lyons, M. J., & Kremen, W. S. (2020). Internalizing and externalizing psychopathology in middle age: Genetic and environmental architecture and stability of symptoms over 15 to 20 years. Psychological Medicine, 50(9), 1530–1538. 10.1017/S0033291719001533 31258104 PMC6938573

[IMAG.a.1308-b30] Han, Z., Yang, G., Liu, T., Funahashi, S., Zuo, X.-N., & Yan, T. (2025). Enriching population diversity in neuroscience. Science Bulletin, 70(16), 2560–2564. 10.1016/j.scib.2025.03.037 40175181

[IMAG.a.1308-b31] Harms, M. P., Somerville, L. H., Ances, B. M., Andersson, J., Barch, D. M., Bastiani, M., Bookheimer, S. Y., Brown, T. B., Buckner, R. L., Burgess, G. C., Coalson, T. S., Chappell, M. A., Dapretto, M., Douaud, G., Fischl, B., Glasser, M. F., Greve, D. N., Hodge, C., Jamison, K. W.,… Yacoub, E. (2018). Extending the Human Connectome Project across ages: Imaging protocols for the Lifespan Development and Aging projects. Neuroimage, 183, 972–984. 10.1016/j.neuroimage.2018.09.060 30261308 PMC6484842

[IMAG.a.1308-b32] Hildon, Z., Montgomery, S. M., Blane, D., Wiggins, R. D., & Netuveli, G. (2010). Examining resilience of quality of life in the face of health-related and psychosocial adversity at older ages: What is “Right” about the way we age? The Gerontologist, 50(1), 36–47. 10.1093/geront/gnp067 19549715

[IMAG.a.1308-b33] Hoogendam, Y. Y., van der Lijn, F., Vernooij, M. W., Hofman, A., Niessen, W. J., van der Lugt, A., Ikram, M. A., & van der Geest, J. N. (2014). Older age relates to worsening of fine motor skills: A population-based study of middle-aged and elderly persons. Frontiers in Aging Neuroscience, 6, 259. 10.3389/fnagi.2014.00259 25309436 PMC4174769

[IMAG.a.1308-b34] Horga, G., Kaur, T., & Peterson, B. S. (2014). Annual research review: Current limitations and future directions in MRI studies of child- and adult-onset developmental psychopathologies. Journal of Child Psychology and Psychiatry, 55(6), 659–680. 10.1111/jcpp.12185 24438507 PMC4029914

[IMAG.a.1308-b35] Hostinar, C. E., & Cicchetti, D. (2020). Emotion dysregulation and internalizing spectrum disorders. In T. P. Beauchaine & S. E. Crowell (Eds.), The Oxford Handbook of Emotion Dysregulation (pp. 1–45). Oxford University Press. 10.1093/oxfordhb/9780190689285.013.18

[IMAG.a.1308-b36] Insel, T. R., & Quirion, R. (2005). Psychiatry as a clinical neuroscience discipline. JAMA, 294(17), 2221–2224. 10.1001/jama.294.17.2221 16264165 PMC1586100

[IMAG.a.1308-b37] Kebets, V., Holmes, A. J., Orban, C., Tang, S., Li, J., Sun, N., Kong, R., Poldrack, R. A., & Yeo, B. T. T. (2019). Somatosensory-motor dysconnectivity spans multiple transdiagnostic dimensions of psychopathology. Biological Psychiatry, 86(10), 779–791. 10.1016/j.biopsych.2019.06.013 31515054

[IMAG.a.1308-b38] Kessler, R. C. (1997). The prevalence of psychiatric comorbidity. In Treatment strategies for patients with psychiatric comorbidity (pp. 23–48). John Wiley & Sons Inc. 10.1176/ps.49.4.542

[IMAG.a.1308-b39] Krishnan, A., Williams, L. J., McIntosh, A. R., & Abdi, H. (2011). Partial least squares (PLS) methods for neuroimaging: A tutorial and review. Neuroimage, 56(2), 455–475. 10.1016/j.neuroimage.2010.07.034 20656037

[IMAG.a.1308-b40] Kroenke, K., & Price, R. K. (1993). Symptoms in the community: Prevalence, classification, and psychiatric comorbidity. Archives of Internal Medicine, 153(21), 2474–2480. 10.1001/archinte.1993.00410210102011 8215752

[IMAG.a.1308-b41] Kroenke, K., Spitzer, R. L., Williams, J. B., & Löwe, B. (2010). The patient health questionnaire somatic, anxiety, and depressive symptom scales: A systematic review. General Hospital Psychiatry, 32(4), 345–359. 10.1016/j.genhosppsych.2010.03.006 20633738

[IMAG.a.1308-b42] Lachman, M. E. (2004). Development in midlife. Annual Review of Psychology, 55, 305–331. 10.1146/annurev.psych.55.090902.141521 14744218

[IMAG.a.1308-b43] Lang, A. J., & Stein, M. B. (2005). An abbreviated PTSD checklist for use as a screening instrument in primary care. Behaviour Research and Therapy, 43(5), 585–594. 10.1016/j.brat.2004.04.005 15865914

[IMAG.a.1308-b44] Lees, B., Squeglia, L. M., McTeague, L. M., Forbes, M. K., Krueger, R. F., Sunderland, M., Baillie, A. J., Koch, F., Teesson, M., & Mewton, L. (2020). Altered neurocognitive functional connectivity and activation patterns underlie psychopathology in preadolescence. Biological Psychiatry: Cognitive Neuroscience and Neuroimaging, 6(4), 387–398. 10.1016/j.bpsc.2020.09.007 33281105 PMC8426459

[IMAG.a.1308-b45] Lessov-Schlaggar, C. N., Rubin, J. B., & Schlaggar, B. L. (2016). The fallacy of univariate solutions to complex systems problems. Frontiers in Neuroscience, 10, 267. 10.3389/fnins.2016.00267 27375425 PMC4896944

[IMAG.a.1308-b46] Litwin, H. (2012). Physical activity, social network type, and depressive symptoms in late life: An analysis of data from the National Social Life, Health and Aging Project. Aging & Mental Health, 16(5), 608–616. 10.1080/13607863.2011.644264 22296412 PMC3430832

[IMAG.a.1308-b47] Love, N., Ruff, G., & Geldmacher, D. (2015). Social cognition in older adults: A review of neuropsychology, neurobiology, and functional connectivity. Medical and Clinical Reviews, 1, 6. 10.21767/2471-299x.1000006

[IMAG.a.1308-b48] Lyall, D. M., Quinn, T., Lyall, L. M., Ward, J., Anderson, J. J., Smith, D. J., Stewart, W., Strawbridge, R. J., Bailey, M. E. S., & Cullen, B. (2022). Quantifying bias in psychological and physical health in the UK Biobank imaging sub-sample. Brain Communications, 4(3), fcac119. 10.1093/braincomms/fcac119 35651593 PMC9150072

[IMAG.a.1308-b49] Mahdipour, N., Shahnazi, H., Hassanzadeh, A., & Sharifirad, G. (2015). The effect of educational intervention on health promoting lifestyle: Focusing on middle-aged women. Journal of Education and Health Promotion, 4(1), 51. 10.4103/2277-9531.162334 26430678 PMC4579766

[IMAG.a.1308-b50] Menon, B. (2019). Towards a new model of understanding – The triple network, psychopathology and the structure of the mind. Medical Hypotheses, 133, 109385. 10.1016/j.mehy.2019.109385 31494485

[IMAG.a.1308-b51] Menon, V. (2011). Large-scale brain networks and psychopathology: A unifying triple network model. Trends in Cognitive Sciences, 15(10), 483–506. 10.1016/j.tics.2011.08.003 21908230

[IMAG.a.1308-b52] Naik, S., Banerjee, A., Bapi, R. S., Deco, G., & Roy, D. (2017). Metastability in senescence. Trends in Cognitive Sciences, 21(7), 509–521. 10.1016/j.tics.2017.04.007 28499740

[IMAG.a.1308-b53] Nakua, H., Yu, J.-C., Abdi, H., Hawco, C., Voineskos, A., Hill, S., Lai, M.-C., Wheeler, A. L., McIntosh, A. R., & Ameis, S. H. (2024). Comparing the stability and reproducibility of brain-behavior relationships found using canonical correlation analysis and partial least squares within the ABCD sample. Network Neuroscience, 8(2), 576–596. 10.1162/netn_a_00363 38952810 PMC11168718

[IMAG.a.1308-b54] Nuevo, R., Chatterji, S., Verdes, E., Naidoo, N., Arango, C., & Ayuso-Mateos, J. L. (2012). The continuum of psychotic symptoms in the general population: A cross-national study. Schizophrenia Bulletin, 38(3), 475–485. 10.1093/schbul/sbq099 20841326 PMC3329982

[IMAG.a.1308-b55] Park, H.-J., Thapa, N., Bae, S., Yang, J.-G., Choi, J., Noh, E.-S., & Park, H. (2024). Association between physical function, mental function and frailty in community-dwelling older adults: A cross-sectional study. Journal of Clinical Medicine, 13(11), 3207. 10.3390/jcm13113207 38892918 PMC11172678

[IMAG.a.1308-b56] Penfield, W., & Boldrey, E. (1937). Somatic motor and sensory representation in the cerebral cortex of man as studied by electrical stimulation. Brain, 60(4), 389–443. 10.1093/brain/60.4.389

[IMAG.a.1308-b57] Pollak, S. D. (2015). Multilevel developmental approaches to understanding the effects of child maltreatment: Recent advances and future challenges. Developmental Psychopathology, 27(4 Pt 2), 1387–1397. 10.1017/s0954579415000826 PMC4832417 26535932

[IMAG.a.1308-b58] Pradhan, B., Chappuis, F., Baral, D., Karki, P., Rijal, S., Hadengue, A., & Gache, P. (2012). The alcohol use disorders identification test (AUDIT): Validation of a Nepali version for the detection of alcohol use disorders and hazardous drinking in medical settings. Substance Abuse Treatment, Prevention, and Policy, 7, 42. 10.1186/1747-597x-7-42 23039711 PMC3508982

[IMAG.a.1308-b59] Qin, S., Ng, E. K. K., Soon, C. S., Chua, X. Y., Zhou, J. H., Koh, W.-P., & Chee, M. W. L. (2025). Association between objectively measured, multidimensional sleep health and cognitive function in older adults: Cross-sectional wearable tracker study. Sleep Medicine, 132, 106569. 10.1016/j.sleep.2025.106569 40393112

[IMAG.a.1308-b60] Rask-Andersen, M., Karlsson, T., Ek, W. E., & Johansson, Å. (2021). Modification of heritability for educational attainment and fluid intelligence by socioeconomic deprivation in the UK Biobank. American Journal of Psychiatry, 178(7), 625–634. 10.1176/appi.ajp.2020.20040462 33900812

[IMAG.a.1308-b61] Raykov, P. P., Knights, E., Cam, C. A. N., & Henson, R. N. (2024). Does functional system segregation mediate the effects of lifestyle on cognition in older adults? Neurobiology of Aging, 134, 126–134. 10.1016/j.neurobiolaging.2023.11.009 38070445 PMC10789480

[IMAG.a.1308-b62] Roelfs, D., Frei, O., van der Meer, D., Tissink, E., Shadrin, A., Alnaes, D., Andreassen, O. A., Westlye, L. T., & Kaufmann, T. (2023). Shared genetic architecture between mental health and the brain functional connectome in the UK Biobank. BMC Psychiatry, 23(1), 461. 10.1186/s12888-023-04905-7 37353766 PMC10290393

[IMAG.a.1308-b63] Rosenblatt, M., Tejavibulya, L., Sun, H., Camp, C. C., Khaitova, M., Adkinson, B. D., Jiang, R., Westwater, M. L., Noble, S., & Scheinost, D. (2024). Power and reproducibility in the external validation of brain-phenotype predictions. Nature Human Behaviour, 8(10), 2018–2033. 10.1038/s41562-024-01931-739085406

[IMAG.a.1308-b64] Russman Block, S. R., Weissman, D. H., Sripada, C., Angstadt, M., Duval, E. R., King, A. P., & Liberzon, I. (2020). Neural mechanisms of spatial attention deficits in trauma. Biological Psychiatry: Cognitive Neuroscience and Neuroimaging, 5(10), 991–1001. 10.1016/j.bpsc.2019.05.014 31377230

[IMAG.a.1308-b65] Samek, D. R., & Hicks, B. M. (2014). Externalizing disorders and environmental risk: Mechanisms of gene-environment interplay and strategies for intervention. Clinical Practice (London), 11(5), 537–547. 10.2217/cpr.14.47 PMC4255466 25485087

[IMAG.a.1308-b66] Schaefer, A., Kong, R., Gordon, E. M., Laumann, T. O., Zuo, X. N., Holmes, A. J., Eickhoff, S. B., & Yeo, B. T. T. (2018). Local-global parcellation of the human cerebral cortex from intrinsic functional connectivity MRI. Cerebral Cortex, 28(9), 3095–3114. 10.1093/cercor/bhx179 28981612 PMC6095216

[IMAG.a.1308-b67] Schnack, H. G., & Kahn, R. S. (2016). Detecting neuroimaging biomarkers for psychiatric disorders: Sample size matters. Frontiers in Psychiatry, 7, 50. 10.3389/fpsyt.2016.00050 27064972 PMC4814515

[IMAG.a.1308-b68] Senju, A. (2013). Atypical development of spontaneous social cognition in autism spectrum disorders. Brain and Development, 35(2), 96–101. 10.1016/j.braindev.2012.08.002 22964276

[IMAG.a.1308-b69] Sha, Z., Wager, T. D., Mechelli, A., & He, Y. (2019). Common dysfunction of large-scale neurocognitive networks across psychiatric disorders. Biological Psychiatry, 85(5), 379–388. 10.1016/j.biopsych.2018.11.011 30612699

[IMAG.a.1308-b70] Shafiei, G., Esper, N. B., Hoffmann, M. S., Ai, L., Chen, A. A., Cluce, J., Covitz, S., Giavasis, S., Lane, C., Mehta, K., Moore, T. M., Salo, T., Tapera, T. M., Calkins, M. E., Colcombe, S., Davatzikos, C., Gur, R. E., Gur, R. C., Pan, P. M.,… Satterthwaite, T. D. (2025). Reproducible brain charts: An open data resource for mapping brain development and its associations with mental health. Neuron, 113(22), 3758–3779.e3756. 10.1016/j.neuron.2025.08.026 40987284 PMC12950001

[IMAG.a.1308-b71] Shields, A., & Cicchetti, D. (1998). Reactive aggression among maltreated children: The contributions of attention and emotion dysregulation. Journal of Clinical Child and Adolescent Psychology, 27(4), 381–395. 10.1207/s15374424jccp2704_2 9866075

[IMAG.a.1308-b72] Skipsey, K., Burleson, J. A., & Kranzler, H. R. (1997). Utility of the AUDIT for identification of hazardous or harmful drinking in drug-dependent patients. Drug and Alcohol Dependence, 45(3), 157–163. 10.1016/S0376-8716(97)01353-7 9179517

[IMAG.a.1308-b73] Smith, P. J., & Merwin, R. M. (2021). The role of exercise in management of mental health disorders: An integrative review. Annual Review of Medicine, 72, 45–62. 10.1146/annurev-med-060619-022943 PMC8020774 33256493

[IMAG.a.1308-b74] Song, Z., Chen, J., Wen, Z., & Zhang, L. (2020). Abnormal functional connectivity and effective connectivity between the default mode network and attention networks in patients with alcohol-use disorder. Acta Radiologica, 62(2), 251–259. 10.1177/0284185120923270 32423229

[IMAG.a.1308-b75] Spreng, R. N., & Andrews-Hanna, J. R. (2015). The default network and social cognition. Brain Mapping: An Encyclopedic Reference, 3, 165–169. 10.1016/b978-0-12-397025-1.00173-1

[IMAG.a.1308-b76] Sudlow, C., Gallacher, J., Allen, N., Beral, V., Burton, P., Danesh, J., Downey, P., Elliott, P., Green, J., Landray, M., Liu, B., Matthews, P., Ong, G., Pell, J., Silman, A., Young, A., Sprosen, T., Peakman, T., & Collins, R. (2015). UK Biobank: An open access resource for identifying the causes of a wide range of complex diseases of middle and old age. PLoS Medicine, 12(3), e1001779. 10.1371/journal.pmed.1001779 25826379 PMC4380465

[IMAG.a.1308-b77] Sun, L., Qin, W., Liang, X., Wang, C., Men, W., Duan, Y., Fan, X. R., Cai, Q., Qiu, S., Wang, M., Gong, Q., Tian, Y., Liang, P., Liu, Z., Zhang, X., Song, H., Ye, Z., Zhang, P., Dong, Q.,… He, Y. (2025). Population-specific brain charts reveal Chinese-Western differences in neurodevelopmental trajectories. bioRxiv. 10.1101/2025.06.17.659820

[IMAG.a.1308-b78] Sydnor, V. J., Larsen, B., Seidlitz, J., Adebimpe, A., Alexander-Bloch, A. F., Bassett, D. S., Bertolero, M. A., Cieslak, M., Covitz, S., Fan, Y., Gur, R. E., Gur, R. C., Mackey, A. P., Moore, T. M., Roalf, D. R., Shinohara, R. T., & Satterthwaite, T. D. (2023). Intrinsic activity development unfolds along a sensorimotor–association cortical axis in youth. Nature Neuroscience, 26(4), 638–649. 10.1038/s41593-023-01282-y 36973514 PMC10406167

[IMAG.a.1308-b79] Szucs, D., & Ioannidis, J. P. A. (2020). Sample size evolution in neuroimaging research: An evaluation of highly-cited studies (1990–2012) and of latest practices (2017–2018) in high-impact journals. Neuroimage, 221, 117164. 10.1016/j.neuroimage.2020.117164 32679253

[IMAG.a.1308-b80] Tandon, M., Cardeli, E., & Luby, J. (2009). Internalizing disorders in early childhood: A review of depressive and anxiety disorders. Child and Adolescent Psychiatric Clinics of North America, 18(3), 593–610. 10.1016/j.chc.2009.03.004 19486840 PMC3184300

[IMAG.a.1308-b81] Tolomeo, S., & Yu, R. (2022). Brain network dysfunctions in addiction: A meta-analysis of resting-state functional connectivity. Translational Psychiatry, 12(1), 41. 10.1038/s41398-022-01792-6 35091540 PMC8799706

[IMAG.a.1308-b82] Tzourio-Mazoyer, N., Landeau, B., Papathanassiou, D., Crivello, F., Etard, O., Delcroix, N., Mazoyer, B., & Joliot, M. (2002). Automated anatomical labeling of activations in SPM using a macroscopic anatomical parcellation of the MNI MRI single-subject brain. Neuroimage, 15(1), 273–289. 10.1006/nimg.2001.0978 11771995

[IMAG.a.1308-b83] Vossel, S., Geng, J. J., & Fink, G. R. (2014). Dorsal and ventral attention systems: Distinct neural circuits but collaborative roles. Neuroscientist, 20(2), 150–159. 10.1177/1073858413494269 23835449 PMC4107817

[IMAG.a.1308-b84] Weightman, M. J., Air, T. M., & Baune, B. T. (2014). A review of the role of social cognition in major depressive disorder. Frontiers in Psychiatry, 5, 179. 10.3389/fpsyt.2014.00179 25566100 PMC4263091

[IMAG.a.1308-b85] Wilk, P., Ruiz-Castell, M., Stranges, S., Bohn, T., Fagherazzi, G., Nicholson, K., Moran, V., Makovski, T. T., Pi Alperin, M. N., Zeegers, M. P., & Samouda, H. (2024). Relationship between multimorbidity, functional limitation, and quality of life among middle-aged and older adults: Findings from the longitudinal analysis of the 2013–2020 Survey of Health, Ageing, and Retirement in Europe (SHARE). Quality of Life Research, 33(1), 169–181. 10.1007/s11136-023-03508-9 37776401 PMC10784342

[IMAG.a.1308-b86] Wright, A. G. C., Krueger, R. F., Hobbs, M. J., Markon, K. E., Eaton, N. R., & Slade, T. (2013). The structure of psychopathology: Toward an expanded quantitative empirical model. Journal of Abnormal Psychology, 122(1), 281–294. 10.1037/a0030133 23067258 PMC3570590

[IMAG.a.1308-b87] Xia, C. H., Ma, Z., Ciric, R., Gu, S., Betzel, R. F., Kaczkurkin, A. N., Calkins, M. E., Cook, P. A., García de la Garza, A., Vandekar, S. N., Cui, Z., Moore, T. M., Roalf, D. R., Ruparel, K., Wolf, D. H., Davatzikos, C., Gur, R. C., Gur, R. E., Shinohara, R. T.,… Satterthwaite, T. D. (2018). Linked dimensions of psychopathology and connectivity in functional brain networks. Nature Communications, 9(1), 3003. 10.1038/s41467-018-05317-y PMC6070480 30068943

[IMAG.a.1308-b88] Yeo, B. T., Krienen, F. M., Sepulcre, J., Sabuncu, M. R., Lashkari, D., Hollinshead, M., Roffman, J. L., Smoller, J. W., Zöllei, L., Polimeni, J. R., Fischl, B., Liu, H., & Buckner, R. L. (2011). The organization of the human cerebral cortex estimated by intrinsic functional connectivity. Journal of Neurophysiology, 106(3), 1125–1165. 10.1152/jn.00338.2011 21653723 PMC3174820

[IMAG.a.1308-b89] Zehra, A., Lindgren, E., Wiers, C. E., Freeman, C., Miller, G., Ramirez, V., Shokri-Kojori, E., Wang, G.-J., Talagala, L., Tomasi, D., & Volkow, N. D. (2019). Neural correlates of visual attention in alcohol use disorder. Drug and Alcohol Dependence, 194, 430–437. 10.1016/j.drugalcdep.2018.10.032 30502544

[IMAG.a.1308-b90] Zeng, L.-L., Shen, H., Liu, L., Wang, L., Li, B., Fang, P., Zhou, Z., Li, Y., & Hu, D. (2012). Identifying major depression using whole-brain functional connectivity: A multivariate pattern analysis. Brain, 135(5), 1498–1507. 10.1093/brain/aws059 22418737

[IMAG.a.1308-b91] Zhang, Y., Huang, C.-C., Zhao, J., Liu, Y., Xia, M., Wang, X., Wei, D., Chen, Y., Liu, B., Zheng, Y., Wu, Y., Chen, T., Cheng, Y., Xu, X., Gong, Q., Si, T., Qiu, S., Cheng, J., Tang, Y.,…Group, D. I.-M. D. D. W. (2024). Dysfunction in sensorimotor and default mode networks in major depressive disorder with insights from global brain connectivity. Nature Mental Health, 2(11), 1371–1381. 10.1038/s44220-024-00323-0

[IMAG.a.1308-b92] Zhu, X., Cortes, C. R., Mathur, K., Tomasi, D., & Momenan, R. (2017). Model-free functional connectivity and impulsivity correlates of alcohol dependence: A resting-state study. Addiction Biology, 22(1), 206–217. 10.1111/adb.12272 26040546 PMC4669235

[IMAG.a.1308-b93] Zhuo, Z., Chai, L., Wang, Y., Gao, P., Xu, X., Ai, L., Ao, F., Bai, Y., Bai, Y., Cole, J. H., Bao, H., Cai, Q., Cao, J., Chen, F., Chen, F., Chen, K., Chen, Y., Cheng, D., Cui, Z.,… Liu, Y. (2026). Charting brain morphology in international healthy and neurological populations. Nature Neuroscience, 29(2), 420–434. 10.1038/s41593-025-02144-5 41461930

