# Supplementary material for: Functional connectome signature of general psychopathology in middle-aged and older adults: Evidence from multi-cohort, multi-ethnic analyses
Source: Imaging Neurosci (Camb). 2026 Jul 20;4:IMAG.a.1308. doi: 10.1162/IMAG.a.1308 (PMC13386346; doi:10.1162/IMAG.a.1308)
Supplement: Supplementary Material [file IMAG.a.1308_supp.pdf]

Supplement 1. Full description of data preprocessing and additional analyses

UKB Image preprocessing

The additional preprocessing steps used a combination of FSL 5.0.2.2, AFNI 17.0.01 and custom Matlab (R2015b) functions to perform the analyses. First, a 6 mm full-width half-maximum kernel was used to smooth the preprocessed UK Biobank images. The data were normalized to MNI space before band-pass filtered between 0.009 Hz and 0.1 Hz. Participants with mean absolute motion > 3mm and had missing ROI data in the final 420 ROIs investigated were excluded from further analyses.

Mental Health Questionnaire

We excluded participants with cancer, stroke and participants with severe cardiac/ respiratory/ neurological/ psychiatric diseases. The field and illness code used can be found in TableS1. Participants were only included if they answered all 36 chosen questionnaire items (see TableS2). All items corresponded to a field number, except for the number of psychotic events which was calculated as a sum of 4 items/ field numbers. If the total counts exceeded 5 or the participants indicated "Too many to count" for any of the individual items, the sum was set to be 5.

Partial least squares (PLS) correlation

Before entering the data into our PLS models, we regressed out the relevant variables from the simple set of confound as previously published (1). For the FC matrices, we regressed out age at imaging, age squared, sex, the interaction between age and sex, head size, site, head motion (mean relative motion as calculated by FEAT) in resting fMRI, date (duration when the acquisition happened since the acquisitions started) and the date squared. For the behavioral measures, we regressed out age finishing the mental health questionnaire, age squared, sex and the interaction between age and sex.

To further evaluate the stability and consistency of the PLS results, we also split the data in half while maintaining the demographic and behavioral composition. We repeated this procedures 100 times. Following a previous publication (2), we calculated Pearson correlations to assess the similarity between brain and behavior saliences from split halves of each iteration. Higher correlations indicate greater consistency across splits. A Z-test was then applied to the distribution of these correlations for the first 2 latent variables. We noted highly significant

results for the first two latent variables across brain (*Z*-score = 3.12, 5.37) and behavior saliences (*Z*-score = 8.02, 18.9), suggesting stable brain-behavior relationships.

The mental health and brain networks patterns were also comparable with the main findings. As demonstration, here we showed the results of the first iteration of the split half sampling. The details of the participants using can be found in TableS3. We observed that the top two latent variables (LV) were consistently identified to be significant in both samples (FigureS2). The mental health and brain networks patterns were comparable with the main findings (FigureS3). Looking at the PLS results, for dataset 1, the first LV, explaining 25.4% of the overall covariance (p=0.010), represented a general psychopathological factor (FigureS3A). A similar factor was also observed in the validation dataset (27.7%, p=0.003). Individuals with lower psychiatric burden exhibited higher functional connectivity in the somatomotor network, lower functional connectivity in the default mode network, as well as lower connectivity between the default and executive control networks (FigureS3B, C).

The second LV, explaining 13.3% of the overall covariance (p=0.0004) in dataset 1 (FigureS4A), emphasized a distinction between alcohol use disorder and symptoms related to depression and PTSD (FigureS4A). A similar pattern was evident in the validation dataset (13.6%, p<0.0001). Less alcohol use disorder severity but more problems related to depression and PTSD were associated with lower functional connectivity in subcortical regions and between the dorsal and ventral attention networks (FigureS4B, C).

Prediction of longitudinal disease trajectories with ANOVA and Tukey's test

To further study the clinical relevance of the derived brain scores, we assessed the relationship between the brain scores from the first two LVs and future outcomes. A series of analysis of variance (ANOVA) analyses were carried out to compare the first and second latent variables' brain score between the four groups for each disease. The statistics can be found in TableS4. Tukey's tests were carried out to identify specific pairwise differences if the group differences were identified to be significant via ANOVA (TableS5, TableS6). The trajectory of hazardous drinking and the associated brain scores can be visualized in FigureS5.

Longitudinal Prediction Analyses Adjusting for Baseline Symptoms

To further evaluate whether baseline brain scores predicted future psychiatric symptom severity independent of baseline symptom burden, we fitted linear regression models separately for alcohol misuse, anxiety, and depression outcomes. Future symptom scores were modeled as

the dependent variable, while baseline symptom severity was included as a covariate to account for pre-existing psychopathology. Additional covariates included age, sex, quadratic age effects, age-by-sex interaction, and the interval between baseline and follow-up mental health assessments. Brain score latent variables were entered separately into the models. To assess the incremental contribution of brain scores, we conducted nested model comparisons using F-tests, contrasting models with and without the brain score terms.

We noted that the addiction vs. affective factor remained significantly associated with future alcohol misuse severity ($p = 0.0063$), whereas associations with anxiety and depression were not significant, consistent with the main ANOVA findings (**Table S7**). Nested model comparisons using F-tests confirmed a significant improvement in model fit for alcohol misuse only ($F(1, 4479) = 7.48$, $p = 0.0063$; **Table S8**). For the general psychopathology brain scores, the association with future depressive symptoms showed a trend-level effect ($p = 0.058$; **Table S7**), accompanied by a trend-level improvement in model fit in the corresponding F-test ($F(1, 5452) = 3.61$, $p = 0.058$; **Table S8**). These findings are reasonable due to strong relationships between baseline and future symptom burden ($p < 2\times10^{-16}$).

Validation of mental health constructs in HCP-Aging cohort

We looked at the sum of the items from the Achenbach Older Adult Self-Report for participants older than 60 years old (3) as an additional validation. In total we included 321 participants (74.53±9.53 years, 147 males) with no missing behavioral and fMRI data. We noted that only the general psychopathology projected brain scores were predictive of mental health outcomes ($r = 0.11$, $p = 0.046$) unlike the addiction vs. ($r = -0.04$, $p = 0.509$), similar to middle-aged adults.

SG70 Acquisition and Image preprocessing

All participants provided informed consent, and the protocol was approved by the Institutional Review Board of the National University of Singapore. All images were acquired on a Siemens MAGNETOM Prisma Fit 3T scanner using a 64-channel head coil. The imaging protocol included: (1) a high-resolution T1-weighted Magnetization Prepared Rapid Gradient Echo (MPRAGE) sequence (voxel size = $1.0 \times 1.0 \times 1.0$ mm^3, repetition time (TR) = 2200 ms, echo time (TE) = 2.45 ms, inversion time = 900 ms, flip angle = 8°, GRAPPA acceleration = 2); (2) a multiband, multi-echo resting-state fMRI sequence (voxel size = $3.0 \times 3.0 \times 3.0$ mm^3, TR = 1000 ms, TEs = 12/29.75/47.5 ms, flip angle = 50°, multiband factor = 4, GRAPPA = 2).

Functional MRI preprocessing was performed using the CBIG preprocessing pipeline, incorporating FreeSurfer 5.3.0, FSL 5.0.10, AFNI version 2011-12-21-1014, ANTs 2.2.0, and custom MATLAB (R2018b) functions, following established procedures (4). The first four frames were discarded to allow for magnetization stabilization. Slice-timing correction was conducted separately for each echo using FSL, followed by motion correction and outlier detection. Spatial distortion correction was applied using opposite phase-encoded field maps acquired in anterior–posterior and posterior–anterior directions. Multi-echo denoising was then performed using the known echo times. Structural-functional alignment was achieved via FreeSurfer's boundary-based registration. Subsequent nuisance regression removed whole-brain, white-matter, cerebrospinal fluid, and 12 motion parameters, followed by linear detrending and censoring of high-motion volumes. The resulting time series were band-pass filtered between 0.009 Hz and 0.08 Hz. Data were then projected onto the fsaverage6 cortical surface and smoothed with a 6 mm full-width at half-maximum Gaussian kernel. Finally, the preprocessed functional images were nonlinearly registered to the MNI152 2 mm standard space.

T1-weighted and fMRI images were quality controlled using visual inspection. Scans were rated as pass, questionable, or fail based on registration quality, image and motion artifacts, and registration accuracy, with any critical failures flagged for exclusion from analyses.

TableS1. List of Data field and Coding used as exclusion criteria

Field number	*Coding*	*Description*	*Domain*
20001	All	cancer code, self-reported	Cancer
2453	NA	cancer diagnosed by doctor	
4056	NA	age diagnosed with stroke	Stroke
20002	1112	chronic obstructive airways disease/copd	Non-cancer Illnesses
20002	1113	emphysema/chronic bronchitis	
20002	1114	bronchiectasis	
20002	1115	interstitial lung disease	
20002	1124	respiratory failure	
20002	1263	dementia	
20002	1262	parkinsons	
20002	1491	brain haemorrhage	
20002	1245	brain abscess	
20002	1425	aneurysm	
20002	1492	aneurysm (aortic)	
20002	1591	aortic aneurysm rupture	
20002	1433	cerebral palsy	
20002	1266	head injury	
20002	1244	nervous system infection	
20002	1240	head or neurological injury	
20002	1469	post-traumatic stress disorder	
20002	1583	ischaemic stroke	
20002	1081	stroke	
20002	1082	Ischemic attack	
20002	1284	other chronic degenerative neurological problems	
20002	1397	demyelinating diseases	
20002	1243	psychological or psychiatric problems	
20002	1286	depression	
20002	1287	anxiety/panic attacks	
20002	1288	nervous breakdown	
20002	1289	schizophrenia	
20002	1291	mania/bipolar disorder/manic depression	
20002	1083	haematoma	
20002	1246	encephalitis	
20002	1086	haematoma haemorrhage	
20002	1408	alcohol dependency	
20002	1409	opioid dependency	
20002	1410	any dependency	
20002	1261	multiple sclerosis	
20002	1264	epilepsy	
20002	1075	heart attack/myocardial infarction	
20002	1074	angina	
20002	1076	heart failure/pulmonary odema	

TableS2. List of Online Mental Health Questionnaire items investigated.

Field number	*Description*	*Domain*
20414	Drink frequency	Alcohol use disorder
20403	Number of drinks	Alcohol use disorder
20416	Excessive drinking	Alcohol use disorder
20413	Unable to stop drinking	Alcohol use disorder
20407	Dysfunction due to drinking	Alcohol use disorder
20412	Reliance on drinks	Alcohol use disorder
20409	Guilt due to drinking	Alcohol use disorder
20408	Forgetfulness from drinking	Alcohol use disorder
20411	Injury from drinking	Alcohol use disorder
20405	Told to reduce drinking	Alcohol use disorder
20506	Feeling nervous, anxious or on edge	Anxiety
20509	Not being able to stop or control worrying	Anxiety
20520	Worrying too much about different things	Anxiety
20515	Trouble relaxing	Anxiety
20516	Being so restless that it is hard to sit still	Anxiety
20505	Becoming easily annoyed or irritable	Anxiety
20512	Feeling afraid as if something awful might happen	Anxiety
20514	Little interest or pleasure in doing things	Depression
20510	Feeling down, depressed or hopeless	Depression
20517	Trouble sleeping	Depression
20519	Feeling tired	Depression
20511	Poor appetite or overeating	Depression
20507	Feeling bad about yourself	Depression
20508	Trouble concentrating	Depression/PTSD
20518	Moving or speaking slowly or fidgety or restless	Depression
20513	Thoughts that you would be better off dead	Depression
20497	Repeated disturbing thoughts of stressful experience past month	PTSD
20498	Upset when reminded of stressful experience in past month	PTSD
20495	Avoided activities/ situations due to stressful experience past month	PTSD
20496	Felt distant from other people in past month	PTSD
20494	Felt irritable or had angry outbursts in past month	PTSD
20468	Ever believed in an un-real conspiracy against self	Psychosis
20474	Ever believed in un-real communications or signs	Psychosis
20463	Ever heard an un-real voice	Psychosis
20471	Ever seen an un-real vision	Psychosis
20470, 20476, 20465, 20473	Number of psychotic events	Psychosis

TableS3. Participant Characteristics of 2 data halves in the first iteration.

	Characteristics	*Dataset 1 (n=3265)*	*Dataset 2 (n=3264)*	*P value*
Demographics	Sex, male:female	1405:1860	1509:1855	0.912
	Age at baseline scan, years, mean (SD)	62.87 (7.36)	62.93 (7.61)	0.764
	Age finishing MHQ, years, mean (SD)	61.70 (7.20)	61.77 (7.48)	0.746
	Site (Cheadle:Reading:Newcastle:Bristol)	1967:492:803:3	1966:489:805:4	0.985
MRI	Head Size, mean (SD)	1.30 (0.12)	1.30 (0.12)	0.802
	Mean Relative Motion, mm, mean (SD)	0.12 (0.06)	0.12 (0.05)	0.515
	Time since first acquisition, years, mean (SD)	3.56 (1.56)	3.55 (1.59)	0.920
MHQ measures	AUDIT	6.80 (4.93)	6.74 (4.80)	0.652
	GAD7	3.11 (3.89)	3.16 (3.96)	0.634
	PHQ9	3.78 (4.21)	3.83 (4.31)	0.607
	PCL6	10.12 (3.30)	10.07 (3.28)	0.513
	Psychotic events	0.18 (0.83)	0.18 (0.83)	0.884

Note: AUDIT: Alcohol Use Disorders Identification Test, GAD7: General Anxiety Disorder-7 questions, MHQ: Mental *health questionnaire, PHQ9: Patient Health Questionnaire 9-question version*, *PCL6: Post-traumatic stress disorder Check List – civilian short version. P values are calculated using Chi square for sex and site, t-test for the rest of the variables.*

TableS4. ANOVA Statistic for all comparisons

Disease	*Brain scores*	*DF*	*F-stat*	*P value*	*Adjusted P value*
Depression	LV1	3	16.471	<0.0001	<0.0001
	LV2	3	0.832	0.4759	0.4759
Anxiety	LV1	3	11.651	<0.0001	<0.0001
	LV2	3	1.028	0.3789	0.4547
Hazardous Drinking	LV1	3	6.31	0.0003	0.0004
	LV2	3	17.746	<0.0001	<0.0001

Note: LV1 represents the general psychopathology factor, LV2 represents the addiction vs. affective factor.

TableS5. Posthoc Tukey HSD Statistic for all significant ANOVA comparisons for the general psychopathology factor brain scores

Comparison	Mean diff.	Lower CI	Upper CI	P value	Adjusted p value
**Depression→no depression vs depression →depression**	-0.254	-0.485	-0.024	0.0238	0.052
**No depression→depression vs depression→depression**	-0.391	-0.629	-0.153	0.0001	0.0001
**No depression→no depression vs depression→depression**	-0.44	-0.614	-0.266	<0.0001	<0.0001
**No depression→depression vs depression→no depression**	-0.137	-0.364	0.091	0.4132	0.4958
**No depression→no depression vs depression→no depression**	-0.186	-0.346	-0.026	0.0151	0.0403
**No depression→no depression vs no depression→depression**	-0.049	-0.22	0.121	0.88	0.9377
**Anxiety→no anxiety vs anxiety→anxiety**	-0.084	-0.369	0.202	0.8758	0.9377
**No anxiety→anxiety vs anxiety→anxiety**	-0.254	-0.545	0.037	0.1123	0.2073
**No anxiety→no anxiety vs anxiety→anxiety**	-0.366	-0.602	-0.13	0.0004	0.0017
**No anxiety→anxiety vs anxiety→no anxiety**	-0.17	-0.41	0.07	0.2628	0.3932
**No anxiety→no anxiety vs anxiety→no anxiety**	-0.283	-0.452	-0.113	0.0004	0.0004
**No anxiety→no anxiety vs no anxiety→anxiety**	-0.112	-0.29	0.066	0.3661	0.4624
**HD→no HD vs HD→ HD**	0.015	-0.118	0.147	0.9922	0.9922
**No HD→ HD vs HD→ HD**	-0.227	-0.374	-0.08	0.0004	0.0017
**No HD→no HD vs HD→ HD**	-0.071	-0.162	0.019	0.1785	0.2855
**No HD→ HD vs HD→no HD**	-0.241	-0.411	-0.072	0.0014	0.0049
**No HD→no HD vs HD→no HD**	-0.086	-0.209	0.037	0.2785	0.3932
**No HD→no HD vs no HD→ HD**	0.156	0.017	0.294	0.0202	0.0485

Note: HD: hazardous drinking

TableS6. Posthoc Tukey HSD Statistic for all significant ANOVA comparisons for the addiction vs affective brain scores

Comparison	Mean diff.	Lower CI	Upper CI	P value	Adjusted p value
**HD→no HD vs HD→ HD**	0.088	-0.045	0.22	0.3227	0.4303
**No HD→ HD vs HD→ HD**	0.133	-0.013	0.279	0.0895	0.179
**No HD→no HD vs HD→ HD**	0.249	0.159	0.339	<0.0001	<0.0001
**No HD→ HD vs HD→no HD**	0.046	-0.123	0.214	0.8987	0.9377
**No HD→no HD vs HD→no HD**	0.161	0.038	0.284	0.0041	0.0124
**No HD→no HD vs no HD→ HD**	0.115	-0.022	0.253	0.1362	0.2334

Note: HD: hazardous drinking

TableS7. Longitudinal associations between baseline brain scores and future psychiatric symptoms adjusting for baseline severity

Outcome	*Brain score*	*Estimate*	*Standard error*	*Statistic*	*P value*
AUDIT	LV1	-0.0857	0.0464	-1.85	0.0649
	LV2	-0.128	0.0469	-2.73	0.00627
GAD7	LV1	0.0428	0.0432	0.991	0.322
	LV2	-0.0588	0.0429	-1.37	0.17
PHQ9	LV1	0.0846	0.0445	1.9	0.0576
	LV2	-0.0408	0.0443	-0.92	0.357

Note: LV1 represents the general psychopathology factor, LV2 represents the addiction vs. affective factor. AUDIT: Alcohol Use Disorders Identification Test, GAD7: General Anxiety Disorder-7 questions, PHQ9: Patient Health Questionnaire 9-question version

TableS8. Nested model comparisons evaluating the contribution of brain scores to prediction of future psychiatric symptoms

Outcome	*Brain score*	R^2 *(base)*	R^2 *(full)*	ΔR^2	*F value*	*df1*	*df2*	*P value*
AUDIT	LV1	0.60267	0.60298	3.02E-04	3.40934	1	4479	0.06489
	LV2	0.60267	0.60334	6.62E-04	7.47783	1	4479	0.00627
GAD7	LV2	0.28608	0.28633	2.46E-04	1.87955	1	5454	0.17044
	LV1	0.28608	0.28621	1.28E-04	0.98183	1	5454	0.32179
PHQ9	LV2	0.36883	0.36893	9.80E-05	0.84681	1	5452	0.35749
	LV1	0.36883	0.36924	4.17E-04	3.60568	1	5452	0.05763

Note: Model comparisons were conducted using F-tests, with df1 corresponding to the number of added parameters (brain score; df1 = 1) and df2 representing the residual degrees of freedom of the full model. ΔR^2 reflects the change in explained variance between models. P-values correspond to the F-test for incremental model fit. LV1 represents the general psychopathology factor, LV2 represents the addiction vs. affective factor. AUDIT: Alcohol Use Disorders Identification Test, GAD7: General Anxiety Disorder-7 questions, PHQ9: Patient Health Questionnaire 9-question version

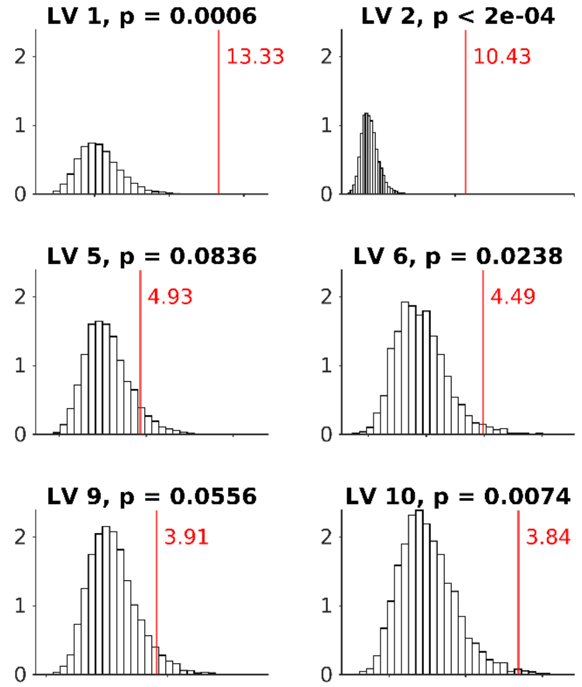

FigureS1. Permutation testing of PLS latent variables. Histograms show the null distributions of singular values for the first ten latent variables (LV1–LV10) obtained from permutation testing (5000 permutations). Distributions are plotted as probability density functions (PDFs), such that the area under each histogram integrates to one. The red vertical line in each panel indicates the singular value from the original (non-permuted) data. Empirical *p*-values are reported for each LV and represent the proportion of permuted singular values greater than or equal to the observed value.

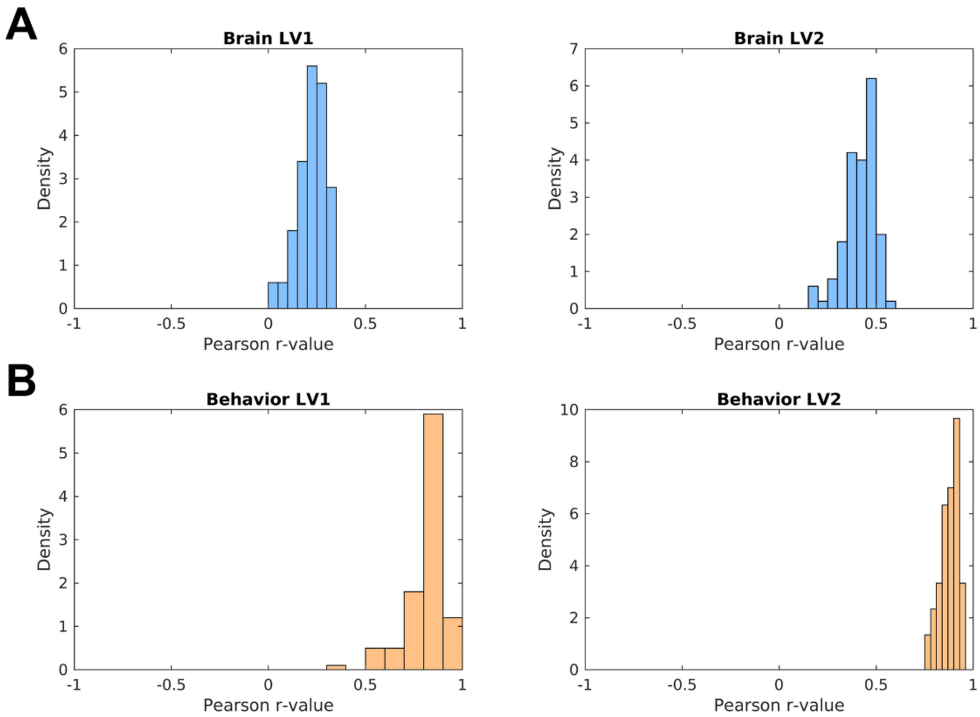

FigureS2. (A) Distributions of the correlations between resampled brain saliences from the split-half analyses for the top two latent variables. **(B)** Distributions of the correlations between resampled behavior saliences from the split-half analyses for the top two latent variables.

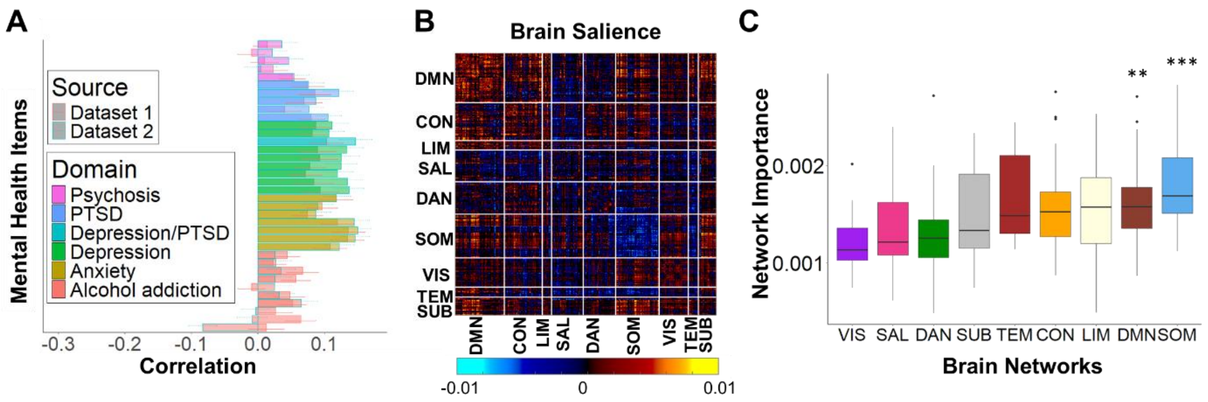

FigureS3. The first latent variable reflects a general psychopathology factor. (A) Correlations of all 36 mental health items with the composite brain scores colored by diagnostic domains. The error bars indicate bootstrapped standard error. **(B)** The shared brain salience showed the average contributions of each connection to the covariance between functional connectivity and the mental health item scores. **(C)** Normalized importance grouped by functional networks reflected the average contribution of each functional network to the latent variable, ordered by median. $^{**}P \leq 0.01$, $^{***}P \leq 0.001$. Abbreviations: PTSD: posttraumatic stress disorder, DMN: Default Mode, CON: Control, LIM: Limbic, SAL: Salience/ Ventral Attention, DAN: Dorsal Attention, SOM: Somatomotor, VIS: Visual, TEM: Temporal, SUB: Subcortical.

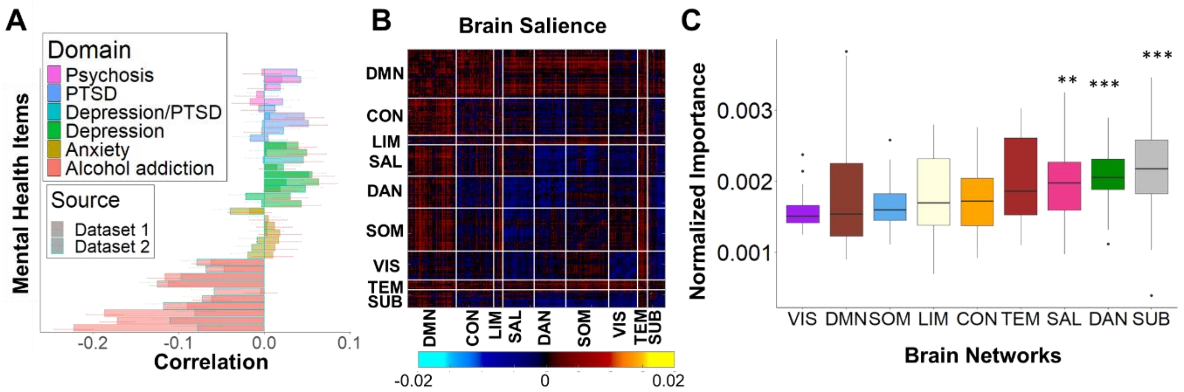

FigureS4. The second latent variable reflects a divergence between alcohol use disorder and depression/PTSD related symptoms. **(A)** Correlations of all 36 mental health items with the composite brain scores colored by diagnostic domains. The error bars indicate bootstrapped standard error. **(B)** The shared brain salience showed the average contributions of each connection to the covariance between functional connectivity and the mental health item scores. **(C)** Normalized importance grouped by functional networks reflected the average contribution of each functional network to the latent variable, ordered by median. $^{**}P \leq 0.01$, $^{***}P \leq 0.001$. Abbreviations: PTSD: posttraumatic stress disorder, DMN: Default Mode, CON: Control, LIM: Limbic, SAL: Salience/ Ventral Attention, DAN: Dorsal Attention, SOM: Somatomotor, VIS: Visual, TEM: Temporal, SUB: Subcortical.

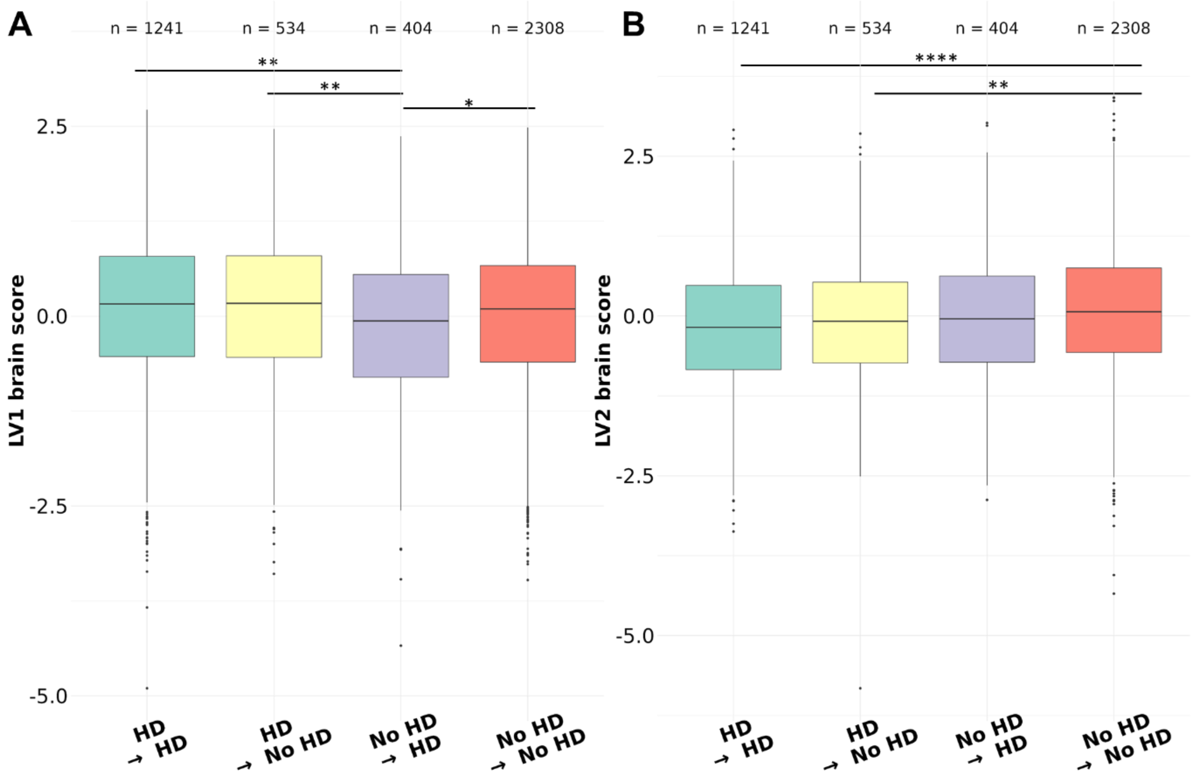

FigureS5. Brain scores differentiate participants with different trajectories of drinking (A) General psychopathology brain score plotted for 4 groups with different current and future hazardous drinking status **(B)** Addiction vs affective brain score plotted for 4 groups with different current and future hazardous drinking status. *Box plots displayed the median and the interquartile range (IQR). The whisker extended from the box to the largest/smallest value no farther than 1.5∗IQR. All values beyond this range were plotted individually. HD: Hazardous drinking.* *$P \leq 0.05$, **$P \leq 0.01$, ***$P \leq 0.001$, **** $P \leq 0.0001$

References:

1. Alfaro-Almagro F, McCarthy P, Afyouni S, Andersson JLR, Bastiani M, Miller KL, et al. Confound modelling in UK Biobank brain imaging. NeuroImage. 2021;224:117002.
2. Nakua H, Yu J-C, Abdi H, Hawco C, Voineskos A, Hill S, et al. Comparing the stability and reproducibility of brain-behavior relationships found using canonical correlation analysis and partial least squares within the ABCD sample. Network Neuroscience. 2024;8(2):576-96.
3. Achenbach TM, Rescorla L. Manual for the ASEBA adult forms & profiles. Burlington, VT: University of Vermont, Research Center for Children, Youth ...; 2003.
4. Li J, Kong R, Liégeois R, Orban C, Tan Y, Sun N, et al. Global signal regression strengthens association between resting-state functional connectivity and behavior. NeuroImage. 2019;196:126-41.
